# ﻿Hidden diversity of *Pestalotiopsis* and *Neopestalotiopsis* (Amphisphaeriales, Sporocadaceae) species allied with the stromata of entomopathogenic fungi in Taiwan

**DOI:** 10.3897/mycokeys.101.113090

**Published:** 2024-01-31

**Authors:** Sheng-Yu Hsu, Yuan-Cheng Xu, Yu-Chen Lin, Wei-Yu Chuang, Shiou-Ruei Lin, Marc Stadler, Narumon Tangthirasunun, Ratchadawan Cheewangkoon, Hind A. AL-Shwaiman, Abdallah M. Elgorban, Hiran A. Ariyawansa

**Affiliations:** 1 Department of Plant Pathology and Microbiology, National Taiwan University, Taipei 106319, Taiwan National Taiwan University Taipei Taiwan; 2 Section of Tea Agronomy, Tea Research and Extension Station, Council of Agriculture, Taoyuan City 326011, Taiwan Council of Agriculture Taoyuan City Taiwan; 3 Department Microbial Drugs, Helmholtz Centre for Infection Research GmbH (HZI), Inhoffenstrasse 7, 38124, Braunschweig, Germany Helmholtz Centre for Infection Research GmbH (HZI) Braunschweig Germany; 4 Department of Biology, School of Science, King Mongkut’s Institute of Technology Ladkrabang (KMITL), Bangkok, 10520, Thailand King Mongkut’s Institute of Technology Ladkrabang (KMITL) Bangkok Thailand; 5 Department of Entomology and Plant Pathology, Faculty of Agriculture, Chiang Mai University, Chiang Mai, 50200, Thailand Chiang Mai University Chiang Mai Thailand; 6 Department of Botany and Microbiology, College of Sciences, King Saud University, P.O. Box 2455, Riyadh, 11451, Saudi Arabia King Saud University Riyadh Saudi Arabia

**Keywords:** DNA sequence data, new species, *Pestalotiopsis* *sensu lato*, taxonomy

## Abstract

*Pestalotiopsissensu lato*, commonly referred to as pestalotiopsis-like fungi, exhibit a broad distribution and are frequently found as endophytes, saprobes and pathogens across various plant hosts. The taxa within pestalotiopsis-like fungi are classified into three genera viz. *Pestalotiopsis*, *Pseudopestalotiopsis* and *Neopestalotiopsis*, based on the conidial colour of their median cells and multi-locus molecular phylogenies. In the course of a biodiversity investigation focusing on pestalotiopsis-like fungi, a total of 12 fungal strains were identified. These strains were found to be associated with stromata of *Beauveria*, *Ophiocordyceps* and *Tolypocladium* in various regions of Taiwan from 2018 to 2021. These strains were evaluated morphologically and multi-locus phylogenetic analyses of the ITS (internal transcribed spacer), *tef1-α* (translation elongation factor 1-α) and *tub2* (beta-tubulin) gene regions were conducted for genotyping. The results revealed seven well-classified taxa and one tentative clade in *Pestalotiopsis* and *Neopestalotiopsis*. One novel species, *Pestalotiopsismanyueyuanani* and four new records, *N.camelliae-oleiferae*, *N.haikouensis*, *P.chamaeropis* and *P.hispanica*, were reported for the first time in Taiwan. In addition, *P.formosana* and an unclassified strain of *Neopestalotiopsis* were identified, based on similarities of phylogeny and morphology. However, the data obtained in the present study suggest that the currently recommended loci for species delimitation of pestalotiopsis-like fungi do not deliver reliable or adequate resolution of tree topologies. The *in-vitro* mycelial growth rates of selected strains from these taxa had an optimum temperature of 25 °C, but growth ceased at 5 °C and 35 °C, while all the strains grew faster under alkaline than acidic or neutral pH conditions. This study provides the first assessment of pestalotiopsis-like fungi, associated with entomopathogenic taxa.

## ﻿Introduction

Fungi are ubiquitous and essential components of all ecosystems on Earth and are more significant to human lives than people assume. Fungi interact with various organisms, including different groups of fungi, to acquire nutrients to successfully complete their life cycle (Ariyawansa et al. 2018b; Tsai et al. 2018; Sun et al. 2019; Tsai et al. 2021; Hsu et al. 2022). In recent years, various kinds of fungi have been identified in different ecological niches, but their ecological roles are largely unknown (Grossart et al. 2019).

Sordariomycetes is one of the largest classes of Ascomycota with members occupying the most varied habitats and niches. The genus *Pestalotiopsis* was initially identified by Steyaert (1949) to accommodate taxa with 5-celled conidia (Liu et al. 2019). Members of this fungal group were traditionally identified, based on the colour density of median conidial cells, apical appendages and conidiogenous cells (Guba 1961; Maharachchikumbura et al. 2014). However, Maharachchikumbura et al. (2014) divided *Pestalotiopsis* into three genera viz. *Pestalotiopsis*, *Pseudopestalotiopsis* and *Neopestalotiopsis*, based on the results of multi-locus phylogenies inferred using the internal transcribed spacer (ITS), β-tubulin (*tub2*) and partial translation elongation factor 1-α (*tef1-α*) gene regions, coupled with morphological features and regarded it as *Pestalotiopsissensu lato* (also known as pestalotiopsis-like fungi). These species are widely distributed in tropical and temperate regions (Maharachchikumbura et al. 2014). Pestalotiopsis-like fungi commonly occur on living plants as pathogens and endophytes or are saprobic on dead plant materials (Maharachchikumbura et al. 2011). Some species of pestalotiopsis-like fungi have been reported as mycoparasites (Xie et al. 2014; Li et al. 2017), while several taxa have been identified as human and insect pathogens (Lv et al. 2011; Monden et al. 2013).

Taiwan is an island in the western Pacific Ocean. The rich diversity of fungal taxa (over 6,670 fungal species) in Taiwan has been frequently reported (Chen et al. 2021; Chen et al. 2022; Hsiao et al. 2022; Huang and Chien 2022). We have been studying pestalotiopsis-like fungi in Taiwan with the aim of providing a natural classification and determining their ecological roles (Ariyawansa et al. 2018b; Tsai et al. 2018; Sun et al. 2019; Tsai et al. 2021; Hsu et al. 2022). However, no published studies have investigated species of pestalotiopsis-like fungi allied with entomogenous fungi. During our investigation of the biodiversity of pestalotiopsis-like fungi in Taiwan, we identified a total of 12 fungal conidia with morphology similar to pestalotiopsis-like fungi. These were found associated with stromata of entomopathogenic fungi. Therefore, the primary objective of this study was to determine the identification and placement of these fungi in *Pestalotiopsissensu lato*, based on DNA sequence-based phylogeny coupled with morphological data. Furthermore, to gain a better understanding of their biology, experiments were conducted to determine the optimal temperature and pH conditions required for mycelial growth of the isolated fungal strains under laboratory conditions.

## ﻿Materials and methods

### ﻿Sample collection, fungal isolation and morphological examination

**Figure 1.**
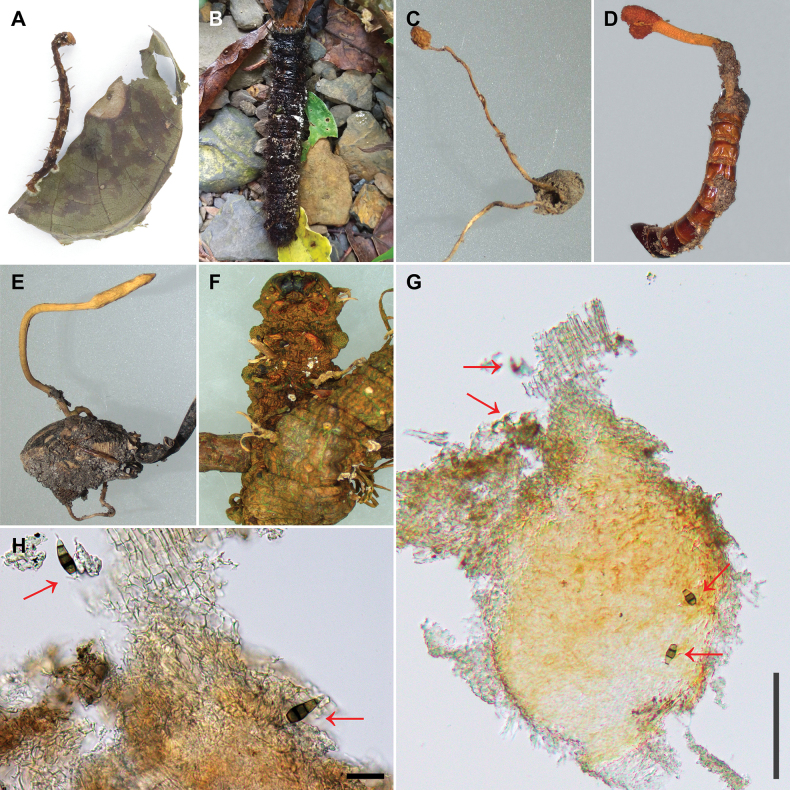
The habitat of *Pestalotiopsis* and *Neopestalotiopsis* species, situated on the stromata of entomopathogenic fungi. Specimens **A***Ophiocordyceps* sp. NTUPPMH 18-160 **B***Beauveria* sp. NTUPPMH 18-161 **C***Tolypocladium* sp. NTUPPMH 21-055 **D***Ophiocordyceps* sp. NTUPPMH 21-054 **E***Ophiocordyceps* sp. NTUPPMH 21-053 **F***Ophiocordyceps* sp. NTUPPMH 18-164 **G, H** the section of conidioma of *Ophiocordyceps* sp. showing the location of conidia of pestalotiopsis-like fungi (red arrow). Scale bars: 100 μm (**G**); 20 μm (**H**).

During an exploration of pestalotiopsis-like fungi between 2018 and 2021 in Taiwan (including Hsinchu County, New Taipei City, Pingtung County, Taichung City, Taoyuan City and Yilan County), fungal spores that are morphologically similar to pestalotiopsis-like fungi were observed on stromata of entomopathogenic fungal species (Fig. 1J, K). The stromata of entomopathogenic fungal species with spores of pestalotiopsis-like fungi were initially mounted in distilled water and separated spores were isolated using the single spore isolation technique as detailed in Choi et al. (1999).

To further study the morphological features of the isolated pestalotiopsis-like fungi, strains were first inoculated on carnation-leaf agar (CLA) (sterile carnation leaf placed on 2% water agar) and incubated at 25 °C with blue light exposure to induce sporulation (Strobel et al. 1996). The conidiomata were placed on a slide and observed through an optical microscope (Olympus DP27) with a digital camera (Olympus BX51). Conidia were imaged and measured with cellSense Standard software (Olympus); 30 measurements were performed for each structure and are shown in Suppl. material 1: table S1.

### ﻿DNA extraction, PCR and sequencing

Isolates were inoculated on potato dextrose agar (PDA) media and incubated at 25 °C in the dark for seven days. Fresh mycelia were harvested and the genomic DNA were extracted from fresh mycelium using EasyPure Genomic DNA Spin Kit (Bioman), following the manufacturer’s protocol (Bioman Scientific Co., Ltd).

PCR amplification was carried out in a 25 μl reaction containing 12.5 μl of 2× Taq Mix-RED (Bioman), 9.5 μl of ddH_2_O, 1 μl of each forward and reverse primer and 1 μl of fungal DNA. Three DNA loci used previously for characterisation of pestalotiopsis-like fungi were selected: ITS, *tub2* and *tef1-α*. Primer sets and touchdown PCR conditions used to amplify ITS, *tub2* and *tef1-α* gene loci are listed in Suppl. material 1: table S4.

### ﻿Strain selection, sequence alignment and phylogenetic analyses

Newly-generated sequence data in this study were observed and manually adjusted via BioEdit version 7.2.5 (Hall et al. 2011; http://bioedit.software.informer.com/) to check the quality of the sequences. Additional related sequences of pestalotiopsis-like fungi were downloaded from GenBank (https://www.ncbi.nlm.nih.gov/nuccore) based on recent publications (Solarte et al. 2018; Fiorenza et al. 2022; Jiang et al. 2022a, 2022b; Peng et al. 2022; Tian et al. 2022; Xiong et al. 2022; Zhang et al. 2022; Guterres et al. 2023; Sun et al. 2023) and are listed in Suppl. material 1: tables S2, S3. Multiple sequence alignments were carried out using MAFFT version 7 (http://mafft.cbrc.jp/alignment/server/index.html) with default settings. To identify closely-associated taxa, single gene phylogenies were inferred for ITS, *tub2* and *tef1-α*, then sequences of these loci were concatenated to conduct a multi-locus analysis including Maximum Likelihood (ML), Maximum Parsimony (MP) and Bayesian Inference (BI) methods.

For the ML analysis, the best-fit substitution models (Suppl. material 1: table S5) were executed for each gene region under the Akaike Information Criterion (AIC) with the nexus-formed partition file from the Model Selection of the IQ-TREE web server (Trifinopoulos et al. 2016; http://iqtree.cibiv.univie.ac.at/). ML trees were inferred with 1,000 bootstrap tests using the ultrafast algorithm in the IQ-TREE and Maximum Likelihood bootstrap (MLB) values ≥ 70% were indicated at each node. MP phylogenetic trees were inferred using the heuristic search option with 1,000 random sequence additions via PAUP version 4.0a169 (Swofford 2003) and Maximum Parsimony bootstrap (MPB) values ≥ 70% were indicated at each node of the final tree obtained from MP analysis.

For the BI analyses, the best evolutionary model was decided under the AIC via MrModelTest version 2.3 (Nylander 2004) and shown in Suppl. material 1: table S5. MrBayes version 3.2.5 (Ronquist et al. 2012) was used to generate Bayesian phylogenetic trees under optimal criteria per partition and posterior probabilities (PP) were determined by Markov Chain Monte Carlo (MCMC) sampling methods. MCMC analysis settings followed previous studies (Tsai et al. 2018, 2021) and four simultaneous Markov chains were initially run for 100,000,000 generations and for every 1000^th^ generation, a tree was sampled (critical value for the topological convergence diagnostic set to 0.01, options of “Bstoprule = yes” and “Bstopval = 0.01”); the MCMC heated chain was set with a “temperature” value of 0.15. The distribution of log-likelihood scores was examined using the Tracer 1.7 programme to determine the stationary phase for each search and to decide whether extra runs were required to achieve convergence (Rambaut et al. 2018). All sampled topologies beneath the asymptote (20%) were discarded as part of a burn-in procedure and the remaining trees were used to calculate PPs in the majority rule consensus tree. All phylogenetic trees and related data files were examined and visualised using FigTree version 1.4.4 (http://tree.bio.ed.ac.uk/software/figtree) and modified using Adobe Illustrator version cc 2022 (https://www.adobe.com/tw/products/illustrator.html).

### ﻿Species delimitation analyses

To determine species delimitations in pestalotiopsis-like fungi, Genealogical Concordance Phylogenetic Species Recognition (GCPSR) was applied (Dettman et al. 2003). Based on the GCPSR principle, species should be recognised when they satisfy one criterion of genealogical concordance or genealogical non-discordance (Dettman et al. 2003; Tsai et al. 2018, 2021). Genealogical concordance was determined if phylogenetic clades were present within at least some gene trees; non-discordance was acknowledged if phylogenetic clades had strong statistical support (MLB ≥ 70%; MPB ≥ 70%; PP ≥ 0.95) in a single locus without conflict at or above this supportive level in any other single-gene trees (Tsai et al. 2018, 2021).

To infer recombination within novel pestalotiopsis-like fungi, the pairwise homoplasy index test (PHI, Ф_w_) (Bruen 2005) and phylogenetic network analysis (Hilário et al. 2021a, 2021b) were employed. The PHI test was used to select hypothesised “species”/populations to infer the occurrence of sexual recombination, based on the standard of incongruence amongst individual single-gene lineages to deduce the recombination level within the complex and between every pair of clades via SplitsTree version 4.16.1 (Huson and Bryant 2006; Hilário et al. 2021b). Results of the PHI index were considered to demonstrate significant recombination occurring with a threshold below 0.05 (Ф_w_ < 0.05). Phylogenetic network analyses were implemented using the LogDet transformation and the NeighborNet algorithm options in SplitsTree software to visualise the relationships between closely-related taxa (Hilário et al. 2021a, 2021b).

### ﻿Mycelial growth test

In total, eight strains representing eight taxa identified in this study were selected to determine the growth rate of mycelia. A 4 mm-diam. mycelial disc was aseptically excised from the edge of the culture and placed at the centre of a PDA media (12 ml in a 9 mm diam. Petri dish). After incubation at 25 °C in the dark for seven days, the diameter of the cultures was measured and two independent tests were conducted with five replicates per trial.

### ﻿Temperature and pH effects on mycelial growth

The same fungal strains, inoculation method and measurement standards used in the mycelial growth test were also used to evaluate the effects of temperature and pH on fungal growth.

Further details for each assessment are described below. The effect of temperature on radial mycelial growth was measured on the seventh day after inoculation at 5, 10, 15, 20, 25, 30, 35 and 40 °C in the dark. All single inoculations were conducted on Petri dishes of 12 ml PDA media. The test was performed twice with five replicates per trial.

The optimal pH for mycelial growth was tested at pH 3, 5, 7, 9 and 11. The PDA plates were heated prior to sterilisation and the pH values were adjusted with 1 M hydrochloric acid (HCl) and 1 M sodium hydroxide (NaOH) solutions. The tested cultures were incubated at 25 °C in the dark for seven days and colony sizes were measured. The test was conducted twice with five replicates per trial.

### ﻿Statistical analysis

Data were processed using Microsoft Excel 2021 to compute the mean and standard deviation. Data analysis was performed with SAS University Edition (version 3.8), utilising one-way analysis of variance (ANOVA) and the mean values were compared using Tukey’s HSD (honestly significant difference) test (α = 0.05) following Tsai et al. (2021).

## ﻿Results

### ﻿Fungal observation and isolation

In total, 12 strains of pestalotiopsis-like fungi associated with the stromata of the entomopathogenic fungi were successfully isolated (Suppl. material 1: table S1 and Fig. 1). Pure cultures of these strains were used in subsequent experiments to understand their molecular taxonomy, biology and diversity.

### ﻿Phylogenetic analysis

ITS sequence data were used for initial identification of the genera of pestalotiopsis-like fungal strains in the present study. Based on the ITS sequence results, 12 strains identified in this study were categorised into two pestalotiopsis-like fungal genera: *Pestalotiopsis* and *Neopestalotiopsis*. Subsequently, to determine the phylogenetic placement of these strains, two different datasets were prepared using the concatenated data matrices of ITS, *tub2* and *tef1-α* gene regions to separately represent the phylogenies.

**Figure 2.**
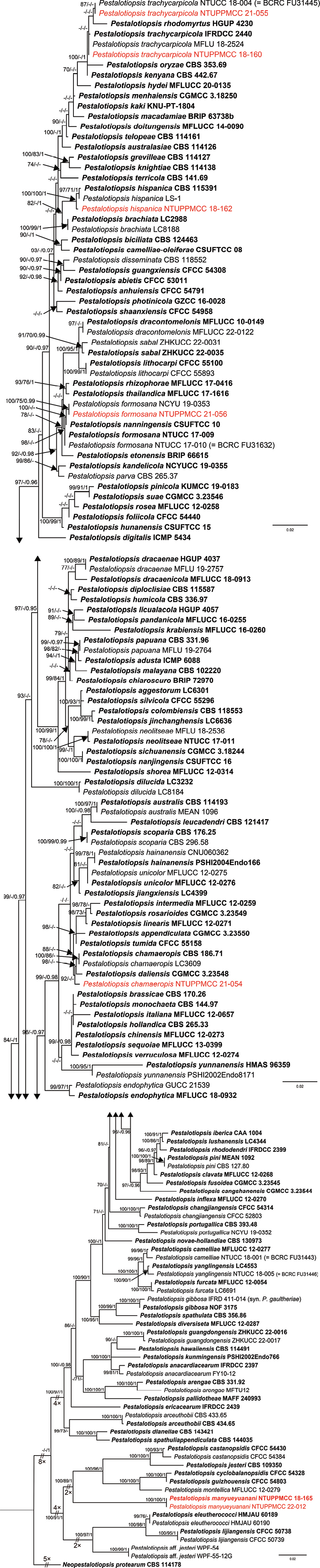
ML phylogenetic tree of *Pestalotiopsis* obtained from the concatenated DNA sequence data of ITS, *tub2* and *tef1-α* genes implemented in IQ-TREE. ML bootstrap values (MLB) ≥ 70%, Maximum Parsimony bootstrap (MPB) values ≥ 70% and Bayesian Posterior Probabilities (PP) ≥ 0.95 are given at the nodes. The scale-bar shows the number of estimated substitutions per site. *Neopestalotiopsisprotearum* (CBS 114178) was used as an outgroup. The new isolates are in red and taxa representing ex-type cultures are in bold.

**Figure 3.**
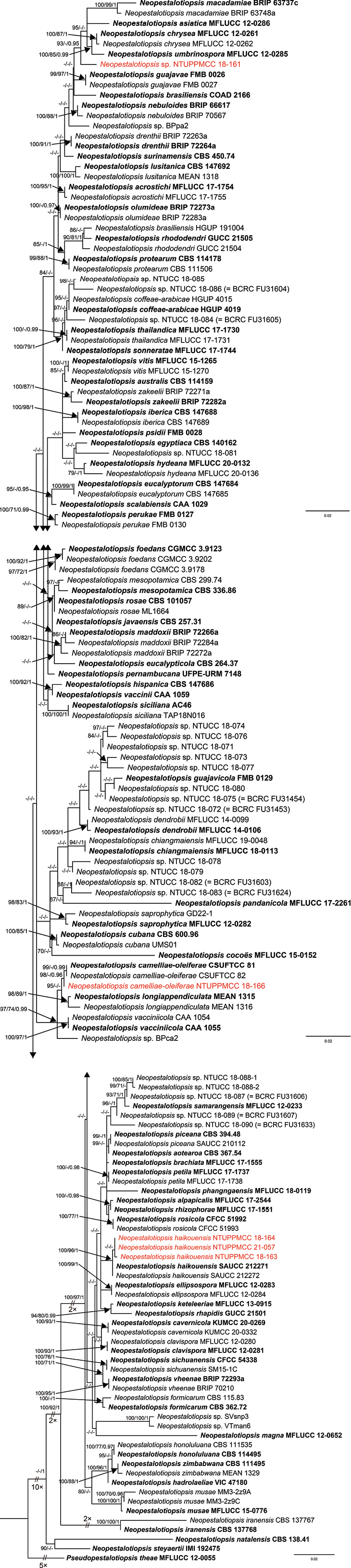
ML Phylogenetic tree of genus *Neopestalotiopsis* attained from the concatenated DNA sequence data of ITS, *tub2* and *tef1-α* loci implemented via IQ-TREE. ML bootstrap values (MLB) ≥ 70%, Maximum Parsimony bootstrap (MPB) values ≥ 70% and Bayesian Posterior Probabilities (PP) ≥ 0.95 are given at the nodes. The scale-bar shows the number of estimated substitutions per site. *Pseudopestalotiopsistheae* (MFLUCC 12-0055) was used as an outgroup. The new isolates are in red and taxa representing ex-type cultures are in bold.

Figs 2, 3 are the phylogenetic trees obtained in different analyses (ML, MP and BI) using concatenated data matrices. In the multi-locus phylogenies, the topologies of the trees inferred for the individual genes were checked visually to confirm that the general tree topologies of the single-gene datasets (Suppl. material 2: figs S5–S10) were similar to each other and resembled the trees obtained from the combined gene dataset alignments.

#### Phylogeny of *Pestalotiopsis*

A total of 160 strains representing 118 accepted species and one unclassified taxon were comprised in the final alignment matrix of *Pestalotiopsis*. *Neopestalotiopsisprotearum* CBS 114178 was assigned as the outgroup taxon (Xiong et al. 2022; Sun et al. 2023). The final dataset was comprised of 1,458 characters (ITS: 550; *tub2*: 457; *tef1-α*: 451), of which 885 characters were constant, 129 variable characters were parsimony-uninformative and 444 were parsimony-informative characters. A best-scoring ML tree resulted in a final ML optimisation value of likelihood of –12523.681; MLB values are presented in Fig. 2. The parsimony analysis of the data matrix resulted in two equally parsimonious trees and the support values of the first tree (tree length, TL = 1,954 steps; consistency index, CI = 0.442; retention index, RI = 0.817; rescaled consistency index, RC = 0.361; homoplasy index, HI = 0.558) and MPB values are presented in Fig. 2. The Bayesian analysis resulted in 69,660 trees after 6,966,000 generations of topological convergence. The first 13,932 trees were discarded, as the burn-in phase of the analyses, whereas the remaining trees were used for computing Bayesian PPs in the majority rule consensus tree, which are shown in Fig. 2. All methods achieved almost the same topology at the species level in compliance with previous studies based on ML, MP and BI (Maharachchikumbura et al. 2014; Tsai et al. 2021; Sun et al. 2023).

Remarkably, two newly-isolated strains in this study (NTUPPMCC 18-165 and 22-012) formed a distinct clade basal to species clades of *P.castanopsidis*, *P.cyclobalanopsidis*, *P.guizhouensis*, *P.jesteri* and *P.montellica* with high statistical support in the single-locus and concatenated data matrices. Thus, the new lineage is introduced as *Pestalotiopsismanyueyuanani* (Fig. 2). A single strain, NTUPPMCC 18-162, was resolved in the clade including the type strain *P.hispanica* (CBS 115391), while one isolate (NTUPPMCC 21-056) clustered within the species clade *P.formosana* (NTUCC 17-009, NTUCC 17-010 and NCYU 19-0353) with high statistical support. Furthermore, the isolate NTUPPMCC 21-054, used in the present study, formed a clade basal to the clade containing the ex-type strains of *P.chamaeropis* (CBS 186.71) and *P.daliensis* (CGMCC 3.23548) with high statistical support in the phylogenetic tree, based on concatenated dataset. The two new isolates (NTUPPMCC 18-160 and NTUPPMCC 21-055), produced in this study, clustered with the strains containing the ex-type strain of *P.trachycarpicola* (IFRDCC 2440) and several representative strains of the species (MFLU 18-2524 and NTUCC 18-004) plus ex-type strains of *P.kenyana* (CBS 442.67), *P.oryzae* (CBS 353.69) and *P.rhodomyrtus* (HGUP 4230) in ITS, *tub2* and multi-locus phylogenies.

#### Phylogeny of *Neopestalotiopsis*

In total, 152 strains representing 74 accepted *Neopestalotiopsis* species constituted the final DNA alignment matrix of *Neopestalotiopsis*. *Pseudopestalotiopsistheae* MFLUCC 12-0055 was used as the outgroup taxon following a recent publication (Guterres et al. 2023). The dataset had 1,440 characters (ITS: 507; *tub2*: 407; *tef1-α*: 523), of which 1,011 characters were constant, 173 variable characters were parsimony-uninformative and 256 were parsimony-informative characters. A best-scoring ML tree resulted in a final ML optimisation value of likelihood of –8052.524 and the final ML tree with MLB values were given in Fig. 3. Parsimony analysis of the data matrix resulted in two equally parsimonious trees and the support values belong to the first tree (TL = 990 steps; CI = 0.547; RI = 0.759; RC = 0.415; HI = 0.453) and the MPB values are shown in Fig. 3. The Bayesian analysis obtained 1,000,000 trees after 100,000,000 generations following topological convergence. The burn-in phase of the analyses showed that the first 20% of trees were discarded and the remaining trees were used for calculating PP in the majority rule consensus tree and the final PP values are plotted in Fig. 3.

However, as mentioned in previous studies and as observed in the present study, the topologies of the *Neopestalotiopsis* phylogenetic trees obtained from all analyses (ML, MP and BI) were unstable and had low statistical support and short branch lengths (Maharachchikumbura et al. 2014; Solarte et al. 2018; Tsai et al. 2021; Fiorenza et al. 2022; Peng et al. 2022; Santos et al. 2022; Zhang et al. 2022). Nevertheless, most of the strains generated in this study formed several consistent clades in both single- and multi-locus analysis. A single isolate (NTUPPMCC 18-166) included in the present study formed a well-supported clade with the ex-type strain of *N.camelliae-oleiferae* CSUFTCC 81, while three isolates (NTUPPMCC 18-163, NTUPPMCC 18-164 and NTUPPMCC 21-057) clustered with the ex-type strain of *N.haikouensis* SAUCC 212271 with robust statistical support. In addition, one isolate (NTUPPMCC 18-161) formed a distinct clade basal to the clades containing ex-type strains of *N.asiatica* (MFLUCC 12-0286), *N.chrysea* (MFLUCC 12-0261), *N.macadamiae* (BRIP 63737c) and *N.umbrinospora* (MFLUCC 12-0285) in multi-locus phylogenetic trees obtained from all analyses (ML, MP and BI). However, the NTUPPMCC 18-161 strain did not consistently form clades in most of the single-locus trees when compared with the results of multi-locus analysis (Suppl. material 2: figs S8–S10). Due to the uncertainty of phylogenetic placement, *Neopestalotiopsis* strain NTUPPMCC 18-161, isolated in this study, was not classified to species level.

### ﻿Taxonomy

#### ﻿ Pestalotiopsis chamaeropis

Taxon classificationFungiAmphisphaerialesSporocadaceae

Maharachch., K.D. Hyde & Crous, 2014

C11C33AE-8D6E-5E83-ADA1-5EFF86A6603C

Fig. 4

##### Description.

On carnation leaves (*Dianthuscaryophyllus*) supplanted on WA (NTUPPMCC 21-054). Sexual morph was not observed in culture. Asexual morph: ***Conidiomata*** acervular, globose, semi-immersed, solitary or gregarious, 200–350 μm diam.; oozing globose, black conidial masses. ***Conidiophores*** obclavate to subcylindrical, 1–3-septate, branched, hyaline, smooth, sometimes merged to conidiogenous cells. ***Conidiogenous cells*** oval to cylindrical or fusiform, hyaline, smooth, (4.8–)5.1–5.7(–5.9) × (20–)21.3–23.9(–25.5) μm, x– ± SD = 5.4 ± 0.3 × 22.6 ± 1.3 μm. ***Conidia*** fusoid, straight or slightly curved, 4-septate, smooth, (2.4–)3.2–5(–5.4) × (4.6–)6.2–10.5(–12.4) μm, x– ± SD = 4.1 ± 0.9 × 8.4 ± 2.2 μm, bearing appendages; basal cell obconic with a truncate base, hyaline, thin-walled, (3.7–)4.2–5.4(–6.2) μm long, x– ± SD = 4.8 ± 0.6 μm; three median cells subcylindrical, pale brown, concolourous, thick-walled, the first median cell from base (3.8–)4.1–4.7(–5) μm long (x– ± SD = 4.4 ± 0.3 μm), the second median cell (4–)4.2–4.8(–5.3) μm long (x– ± SD = 4.5 ± 0.3 μm), the third median cell (3.9–)4.3–5(–5.2) μm long (x– ± SD = 4.6 ± 0.3 μm), together (12–)12.8–14.2(–15.1) μm long (x– ± SD = 13.5 ± 0.7 μm); apical cell conical to subcylindrical with a truncate or acute apex, hyaline, thick-walled, (3.6–)4–4.7(–5.1) μm long (x– ± SD = 4.3 ± 0.4 μm). ***Appendages*** tubular, hyaline, straight or slightly bent, apical appendage 2–3 (mostly 3), unbranched, (8.8–)10.8–17.2(–23.9) μm long (x– ± SD = 14.0 ± 3.2 μm), basal appendage 1–2 (mostly single), centric, unbranched, (2.9–)4.5–7.7(–10.0) μm long (x– ± SD = 6.1 ± 1.6 μm).

**Figure 4.**
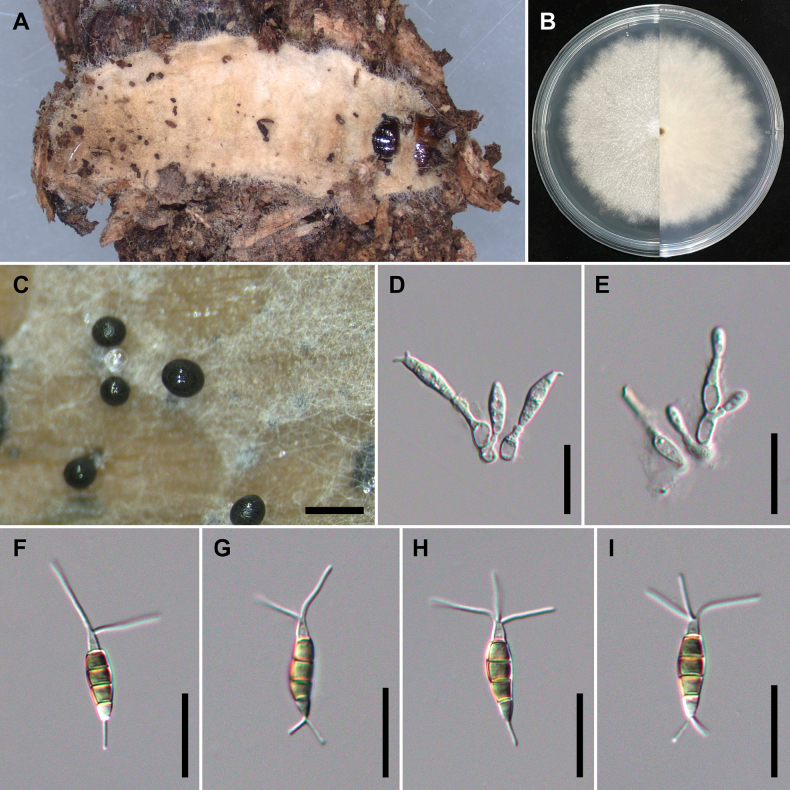
*Pestalotiopsischamaeropis* (NTUPPMCC 21-054 = CD09) **A** the original habitat of *Pestalotiopsischamaeropis*; the stroma of *Ophiocordyceps* sp. **B** top view (left) and bottom view (right) of the colony on potato dextrose agar (PDA) after incubation for seven days **C** conidiomata on carnation leaf **D, E** conidiogenous cells and immature conidia **F–I** conidia. Scale bars: 250 μm (**C**); 20 μm (**D–I**).

##### Culture characteristics.

Colonies on PDA reaching 71.3 mm diam. on average after culturing at 25 °C in the dark for seven days, flat with smooth edge, aerial mycelium dense, white; reverse similar in colour.

##### Materials examined.

Taiwan, Taichung City, Heping District, Yuanzui Mountain, on stroma of *Ophiocordyceps* sp. parasitic on a cocoon (Lepidoptera), 6 July 2021, Ming-Syun Wu, living culture NTUPPMCC 21-054 (= CD09).

##### Notes.

For ML, MP and BI with both single locus and concatenated datasets used in the present study, isolate NTUPPMCC 21-054 formed a clade sister to the clade containing the ex-type strains of *P.chamaeropis* (CBS 186.71) and *P.daliensis* (CGMCC 3.23548) with high statistical support. However, we did not find clear morphological support to consider our isolate as a separate species because NTUPPMCC 21-054 showed overlapping morphologies with both *P.chamaeropis* (CBS 186.71) and *P.daliensis* (CGMCC 3.23548) (Suppl. material 1: table S6). Therefore, giving priority to the oldest name between *P.chamaeropis* and *P.daliensis*, we tentatively named NTUPPMCC 21-054 as *P.chamaeropis* rather than introducing it as a new species. To the best of our knowledge, this is the first report of *P.chamaeropis* in Taiwan.

#### ﻿ Pestalotiopsis formosana

Taxon classificationFungiAmphisphaerialesSporocadaceae

H.A. Ariyaw. & K.D. Hyde, 2018

5B0916F9-EA29-551F-B139-C5E3D7C24F62

Fig. 10

##### Description.

See Suppl. material 1: table S1.

##### Materials examined.

Taiwan, Hsinchu County, Jianshi Township, Ptlaman Mountain, on stroma of *Ophiocordyceps* sp. parasitic on an insect (Coleoptera), 28 July 2021, Li-Hong Chen, living culture NTUPPMCC 21-056 (= CD11).

##### Notes.

Morphological features of *Pestalotiopsisformosana* (NTUPPMCC 21-056), obtained in this study, overlap with the original taxonomic description of *P.formosana* in Ariyawansa and Hyde (2018). Hence, considering both the phylogeny, based on DNA sequence data and morphological characterisation, NTUPPMCC 21-056 was recognised as *Pestalotiopsisformosana*.

#### ﻿ Pestalotiopsis hispanica

Taxon classificationFungiAmphisphaerialesSporocadaceae

F. Liu, L. Cai & Crous

E2B6591A-7548-5500-AC97-5D488193A1F9

Fig. 5

##### Description.

On carnation leaves (*Dianthuscaryophyllus*) supplanted on WA (NTUPPMCC 18-162). Sexual morph was not observed in culture. Asexual morph: ***Conidiomata*** acervular, globose, solitary or gregarious, semi-immersed, 100–500 μm diam.; oozing globose to clavate, black conidial masses. ***Conidiophores*** subcylindrical, hyaline, smooth, annelidic, indistinct and frequently merged to conidiogenous cells. ***Conidiogenous cells*** long pyriform to cylindrical, hyaline, smooth, (1.8–)2.0–3.7(–5.6) × (6.3–)9.7–17.2(–23.2) μm, x– ± SD = 2.9 ± 0.8 × 13.5 ± 3.8 μm. ***Conidia*** fusoid, straight or slightly curved, 4-septate, smooth, (4.7–)5.5–6.5(–7.5) × (21.1–)22.4–25.4(–27.4) μm, x– ± SD = 6.0 ± 0.5 × 23.9 ± 1.5 μm, bearing appendages; basal cell obconic with a truncate base, hyaline or pale brown, thin-walled, (0.8–)4.0–6.1(–6.6) μm long, x– ± SD = 5.0 ± 1.0 μm; three median cells long doliiform to subcylindrical, pale brown, concolourous, thick-walled, the first median cell from base (4.1–)4.5–5.3(–5.8) μm long (x– ± SD = 4.9 ± 0.4 μm), the second median cell (3.8–)4.3–5.0(–5.3) μm long (x–± SD = 4.6 ± 0.3 μm), the third median cell (4.3–)4.6–5.4(–6.3) μm long (x– ± SD = 5.0 ± 0.4 μm), together (12.7–)13.7–15.2 (–16.0) μm long (x– ± SD = 14.5 ± 0.8 μm); apical cell conical to subcylindrical with a truncate or acute apex, hyaline, thick-walled, (3.5–)3.8–4.7(–5.2) μm long (x– ± SD = 4.3 ± 0.5 μm). ***Appendages*** tubular, hyaline, straight or slightly bent, apical appendage 2–3, unbranched, (8.5–)12.1–18.7(–24.9) μm long (x– ± SD = 15.4 ± 3.3 μm), basal appendage 1–3 (mostly single), centric, unbranched (rarely branched), (2.5–)3.2–6.1(–8.8) μm long (x– ± SD = 4.7 ± 1.4 μm).

**Figure 5.**
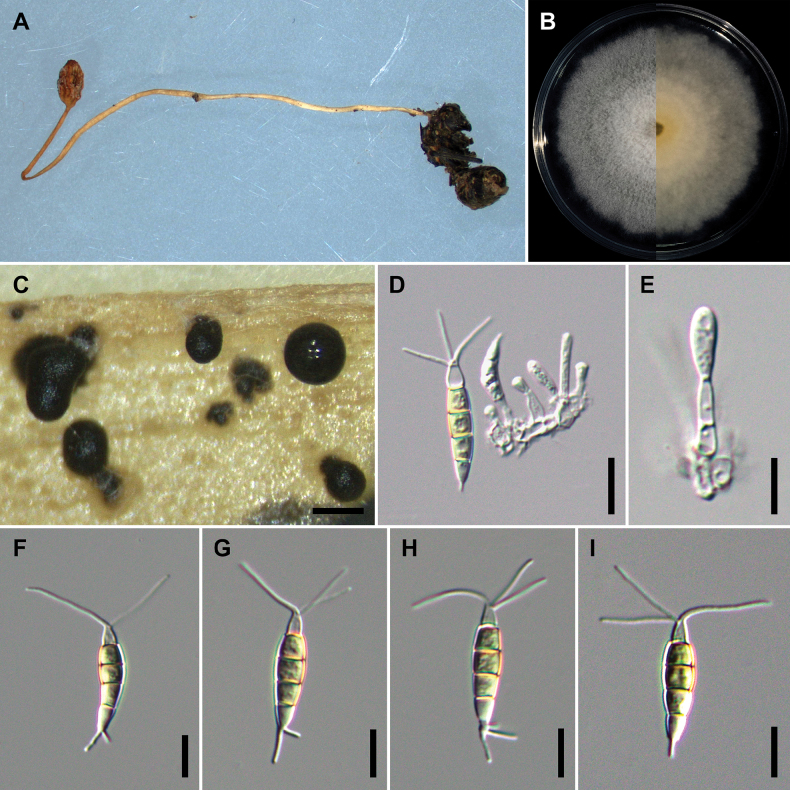
*Pestalotiopsishispanica* (NTUPPMCC 18-162 = CD03) **A** the original habitat of *Pestalotiopsishispanica*; the stroma of *Ophiocordyceps* sp. **B** top view (left) and bottom view (right) of the colony on potato dextrose agar (PDA) after incubation for seven days **C** conidiomata on carnation leaf **D, E** conidiogenous cells and immature conidia **F–I** conidia. Scale bars: 250 μm (**C**); 10 μm (**D–I**).

##### Culture characteristics.

Colonies on PDA reaching 71.9 mm diam. on average after culturing at 25 °C in the dark for seven days, circular, flat with slightly undulate edge, aerial mycelium dense, white; reverse yellowish.

##### Materials examined.

Taiwan, Taoyuan City, Dongyanshan, on the stroma of *Ophiocordyceps* sp. parasitic on an insect (Hymenoptera), 7 October 2018, Wei-Yu Chuang, living culture NTUPPMCC 18-162 (= CD03).

##### Notes.

Multi-locus phylogenetic analysis indicated that strain NTUPPMCC 18-162 was clustered in the same clade with the ex-type strain of *P.hispanica* CBS 115391 with absolute statistical support (MLB = 100%, MPB = 100%, PP = 1.00). Even though the DNA sequences of ITS (100%), *tub2* (99.22%) and *tef1-α* (100%) genes of NTUPPMCC 18-162 were very similar to the ex-type strain of *P.hispanica* (CBS 115391), the morphology of our strain is somewhat different from the original description of *P.hispanica* (holotype CBS H-23554) published by Liu et al. (2019). For instance, NTUPPMCC 18-162 has longer apical appendages (12–18 μm versus 2–14 μm) and contains higher numbers of basal appendages (0–1 versus 0–3) (Suppl. material 1: table S7) compared to the *P.hispanica* holotype CBS H-23554. However, *P.hispanica* was originally isolated from *Protea* cv. ‘Susara’ (Proteaceae) and collected in Spain, while NTUPPMCC 18-162 was isolated from the stroma of *Ophiocordyceps* sp. in Taiwan. Different locations, nutrition modes and natural habitats might have affected the morphology of these two strains. Therefore, considering the identity of the DNA sequences data, we classify NTUPPMCC 18-162 as *P.hispanica* and, to the best of our knowledge, this is the first report of *P.hispanica* in Taiwan.

#### ﻿ Pestalotiopsis manyueyuanani

Taxon classificationFungiAmphisphaerialesSporocadaceae

S.Y. Hsu, Y.C. Xu, W.Y. Chuang & Ariyawansa sp. nov.

19CFA536-5680-55BF-835C-713BDB020024

MycoBank No: 849030

Fig. 6

##### Etymology.

The specific epithet ‘*manyueyuanani*’ is based on the place the fungus was originally collected.

##### Typification.

Taiwan, New Taipei City, Manyueyuan National Forest Recreation Area, on stroma of *Ophiocordyceps* sp. parasitic on an insect (*Cletus* sp., Hemiptera), 25 May 2018, Wei-Yu Chuang, holotype, NTUPPMH 18-165 (permanently preserved in a metabolically inactive state), ex-holotype NTUPPMCC 18-165 (= CD07). *ibid*., ex-isotype NTUPPMCC 22-012.

**Figure 6.**
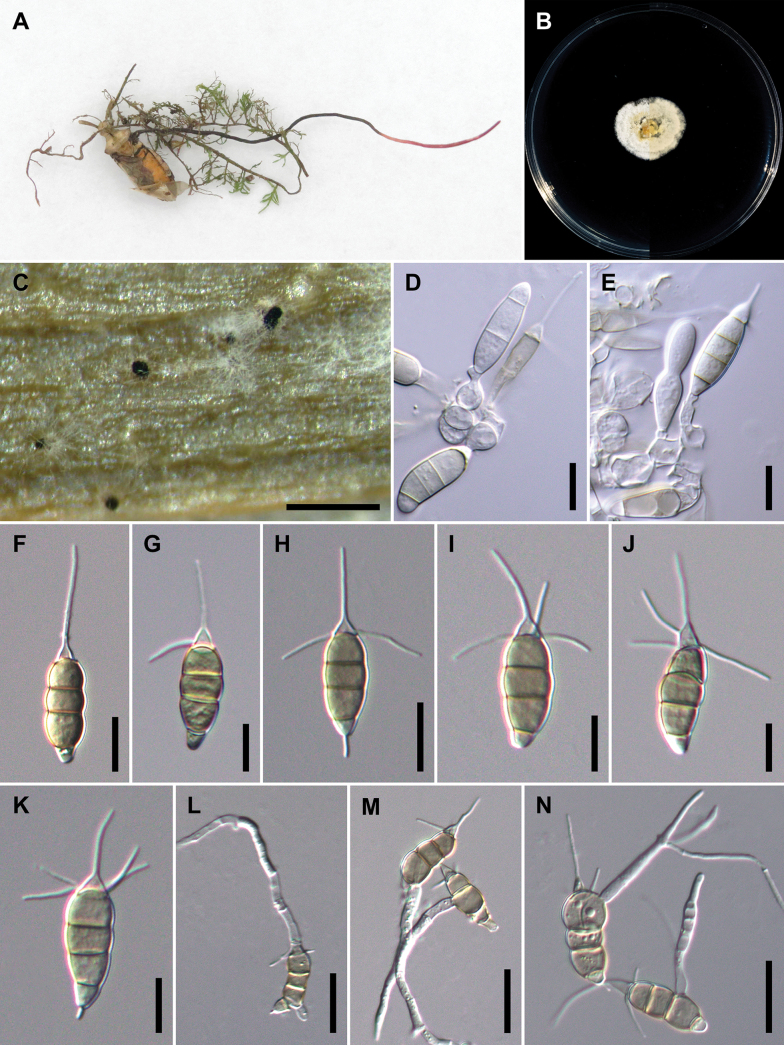
The morphology of *Pestalotiopsismanyueyuanani***A** the original habitat of conidia of *Pestalotiopsismanyueyuanani*; stroma of *Ophiocordyceps* sp. **B** top view (left) and bottom view (right) of the colony on potato dextrose agar (PDA) after incubation for seven days **C** formation of conidiomata on carnation leaf **D, E** conidiogenous cells and immature conidia **F–K** conidia **L–N** germinated conidia. Scale bars: 250 μm (**C**); 10 μm (**D–K**); 20 μm (**L–N**).

##### Description.

Based on the morphology of ex-holotype 18-165 growing on carnation leaves (*Dianthuscaryophyllus*) supplanted on WA. Sexual morph was not observed on culture. Asexual morph: ***Conidiomata*** acervular, globose, scattered, solitary, semi-immersed, black, < 30–100 μm diam.; oozing globose to subcylindrical, black conidial masses. ***Conidiophores*** pyriform to subcylindrical, hyaline, smooth, indistinct and frequently merged to conidiogenous cells. ***Conidiogenous cells*** ampulliform to spherical, hyaline, smooth, (2.8–)3.8–5.3(–6.0) × (6.8–)8.2–12.4(–14.7) μm, x– ± SD = 4.6 ± 0.7 × 10.3 ± 2.1 μm. ***Conidia*** fusoid, straight or slightly curved, 4-septate, smooth, slightly constricted at the septa, (6.7–)7.4–9.2(–10.4) × (22.5–)24.6–30.0(–32.7) μm, x– ± SD = 8.3 ± 0.9 × 27.3 ± 2.7 μm, bearing appendages; basal cell obconic with a truncate base, hyaline or pale brown, thin-walled, (2.9–)3.5–4.7(–5.3) μm long, x– ± SD = 4.1 ± 0.6 μm; three median cells doliiform to subcylindrical, pale brown to brown, concolourous, thick-walled, the first median cell from base (5.3–)5.8–7.9(–9.2) μm long (x– ± SD = 6.9 ± 1.0 μm), the second median cell (4.1–)4.9–6.6(–7.8) μm long (x– ± SD = 5.7 ± 0.9 μm), the third median cell (4.3–)5.5–7.3(–8.6) μm long (x– ± SD = 6.4 ± 0.9 μm), together (15.1–)16.7–21.2 (–24.5) μm long (x– ± SD = 18.9 ± 2.3 μm); apical cell conical with an acute apex, hyaline, thick-walled, (2.2–)3.3–5.1(–5.8) μm long (x– ± SD = 4.2 ± 0.9 μm). ***Appendages*** tubular, hyaline, unbranched, straight or slightly bent, apical appendage single (rarely two), (3.9–)8.7–16.8(–19.1) μm long (x– ± SD = 12.8 ± 4.0 μm), lateral appendages 1–4 (mostly 2, occasionally absent), forming from apical cell, arising above the septum dividing the apical cell and the third median cell, (5.4–)7.3–13.4(–15.4) μm long (x– ± SD = 10.3 ± 3.0 μm), basal appendage single (occasionally absent), centric, (1.8–)2.7–5.5(–6.5) μm long (x– ± SD = 4.1 ± 1.4 μm). Germinating conidia pattern, solitary or multiple, forming from inflated apical cell or median cells.

##### Culture characteristics.

Colonies on PDA reaching 18–24 mm diam. after culturing at 25 °C in the dark for seven days, circular, flat with entire to slightly undulate edge, aerial mycelium sparse, yellowish to orange in the centre, whitish at the margin; reverse similar in colour.

##### Notes.

**Figure 7.**
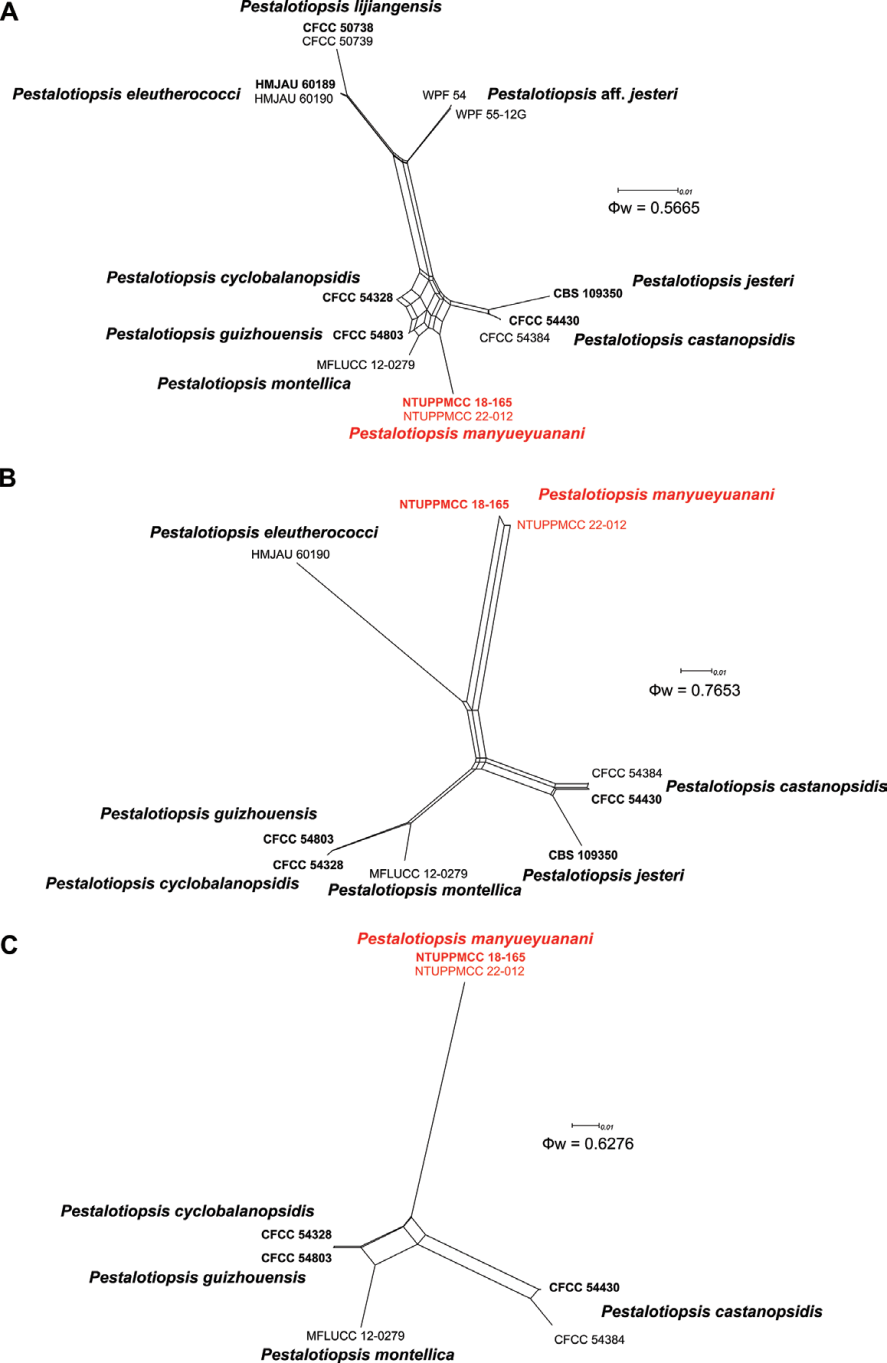
Split graphs showing the results of PHI tests for three gene regions (**A**ITS**B***tub2***C***tef1-α*) of *Pestalotiopsismanyueyuanani* with their phylogenetically closely -related species using LogDet transformation and splits decomposition options. The new taxon in each graph is shown in red and taxa representing ex-type strains are in bold.

*Pestalotiopsismanyueyuanani* sp. nov. is a representative of *Pestalotiopsis* in having pale brown to brown, concolourous median cells without knobbed apical appendages. In both single and concatenated gene analysis, two isolates of *P.manyueyuanani* clustered in a distinct clade with strong statistical support basal to the clade comprising *P.castanopsidis* CFCC 54384 and CFCC 54430, *P.cyclobalanopsidis* CFCC 54328, *P.guizhouensis* CFCC 54803, *P.jesteri* CBS 109350 and *P.montellica* MFLUCC 12-0279 (Fig. 2 and Suppl. material 2: figs S5–S7). However, *P.manyueyuanani* has overlapping conidial morphologies with *P.castanopsidis*, *P.cyclobalanopsidis*, *P.eleutherococci*, *P.guizhouensis*, *P.jesteri*, *P.lijiangensis* and *P.montellica*, showing that these taxa are cryptic species as shown in Suppl. material 1: table S8. At present, the species limitations of cryptic taxa are widely determined by phylogenies, based on single/multi-locus sequence data together with ecology (including host range and pathogenicity), distribution or physiology (Crous et al. 2015; Tsai et al. 2018). Apart from the unique placement in phylogenetic inference, *P.manyueyuanani* differs from other taxa clustered as mentioned above, by host and distribution (Suppl. material 1: table S2). In addition, to further support our hypothesis, we also implemented PHI tests to determine if there are any occurrences of sexual recombination between *P.manyueyuanani* and its closely-related taxa (*P.castanopsidis*, *P.cyclobalanopsidis*, *P.eleutherococci*, *P.guizhouensis*, *P.jesteri*, *P.lijiangensis* and *P.montellica*). The PHI tests indicated that there were no significant recombinations detected within tested groups (Fig. 7, ITS: Ф_w_ = 0.5665; *tub2*: Ф_w_ = 0.7653; *tef1-α*: Ф_w_ = 0.6276), supporting reproductive isolation within the phylogenetically closely-related groups. Therefore, based on these observations, we introduce *P.manyueyuanani* (NTUPPMCC 18-165 and NTUPPMCC 22-012) as a novel species in the genus *Pestalotiopsis*.

#### ﻿ Pestalotiopsis trachycarpicola

Taxon classificationFungiAmphisphaerialesSporocadaceae

Yan M. Zhang & K.D. Hyde, 2012

C0633A13-BD7C-54A8-BB94-CBDAE0C18312

Fig. 10

##### Description:

See Suppl. material 1: table S1.

##### Materials examined.

Taiwan, Yilan County, Yuanshan Township, on stroma of *Ophiocordyceps* sp. parasitic on an insect (Lepidoptera), 15 June 2018, Wei-Yu Chuang, living culture NTUPPMCC 18-160 (= CD01). Taiwan, Taichung City, Heping District, Yuanzui Mountain, on stroma of *Ophiocordyceps* sp. parasitic on an ootheca (Mantodea), 14 July 2021, Ming-Syun Wu, living culture NTUPPMCC 21-055 (= CD10).

##### Notes.

The two new strains NTUPPMCC 18-160 and NTUPPMCC 21-055, used in the present study, clustered within a clade containing ex-type strains of four *Pestalotiopsis* taxa, namely *P.kenyana* (CBS 442.67), *P.oryzae* (CBS 353.69), *P.rhodomyrtus* (HGUP 4230) and *P.trachycarpicola* (IFRDCC 2440) in both single- and multi-locus phylogenies with poor statistical support and short branch lengths. Furthermore, a comparison of the morphological features of these four species and the two strains used in the present study revealed overlapping characteristics, as shown in Suppl. material 1: table S9. However, two strains, included in the present study, tentatively named as *P.trachycarpicola* (Zhang et al. 2012) giving the priority for the oldest species name amongst these four *Pestalotiopsis* species. Nevertheless, further studies of *P.trachycarpicola* (IFRDCC 2440), *P.rhodomyrtus* (HGUP 4230), *P.oryzae* (CBS 353.69) and *P.kenyana* (CBS 442.67) are essential to determine whether these species belong to a single population or if the few informative loci used in the present and previous studies (Liu et al. 2017; Tsai et al. 2021) lead to the poorly-resolved phylogram.

#### ﻿ Neopestalotiopsis camelliae-oleiferae

Taxon classificationFungiAmphisphaerialesSporocadaceae

Qin Yang & He Li, 2021

CA62C2F1-8FCE-5A5C-BCCF-1F453C8F901A

Fig. 8

##### Description.

On carnation leaves (*Dianthuscaryophyllus*) supplanted on WA (NTUPPMCC 18-166). Sexual morph was not observed in culture. Asexual morph: ***Conidiomata*** acervular, globose, semi-immersed, solitary or gregarious, 50–250 μm diam.; oozing globose, black conidial masses. ***Conidiophores*** obclavate to subcylindrical, hyaline, smooth, annelidic, indistinct and frequently merged to conidiogenous cells. ***Conidiogenous cells*** ampulliform to fusiform, hyaline or sometimes pale brown, smooth, (2.1–)2.9–4.5(–5.2) × (3.8–)5.5–8.7(–10.8) μm, x– ± SD = 3.7 ± 0.8 × 7.1 ± 1.6 μm. ***Conidia*** fusoid, straight or slightly curved, 4-septate, smooth, (5.5–)6.3–7.3(–7.8) × (22.1–)23.5–27.4(–29.4) μm, x– ± SD = 6.8 ± 0.5 × 25.4 ± 2 μm, bearing appendages; basal cell obconic with a truncate base, hyaline, thin-walled, (3.6–)4.6–5.8(–6.6) μm long, x– ± SD = 5.2 ± 0.6 μm; three median cells doliiform to subcylindrical, versicoloured, septa darker than the rest of the cell, thick-walled, the first median cell from base pale brown, (4.1–)4.4–5.7(–6.1) μm long (x– ± SD = 5.1 ± 0.6 μm), the second median cell medium to dark brown, (4.1–)4.7–5.9(–6.4) μm long (x– ± SD = 5.3 ± 0.6 μm), the third median cell medium to dark brown, (2.9–)4.6–6.1(–6.6) μm long (x– ± SD = 5.3 ± 0.7 μm), together (13.2–)14.4–17(–18.1) μm long (x– ± SD = 15.7 ± 1.3 μm); apical cell conical to subcylindrical with a truncate or acute apex, hyaline, thick-walled, (3.5–)4.1–5(–5.4) μm long (x– ± SD = 4.5 ± 0.4 μm). ***Appendages*** tubular, hyaline, straight or slightly bent, apical appendage 2–4 (mostly 3), unbranched, (16.3–)21.0–27.0(–30.1) μm long (x– ± SD = 24.0 ± 3.0 μm), basal appendage single, centric, unbranched, (4–)6.5–9.6(–10.3) μm long (x– ± SD = 8.1 ± 1.5 μm).

**Figure 8.**
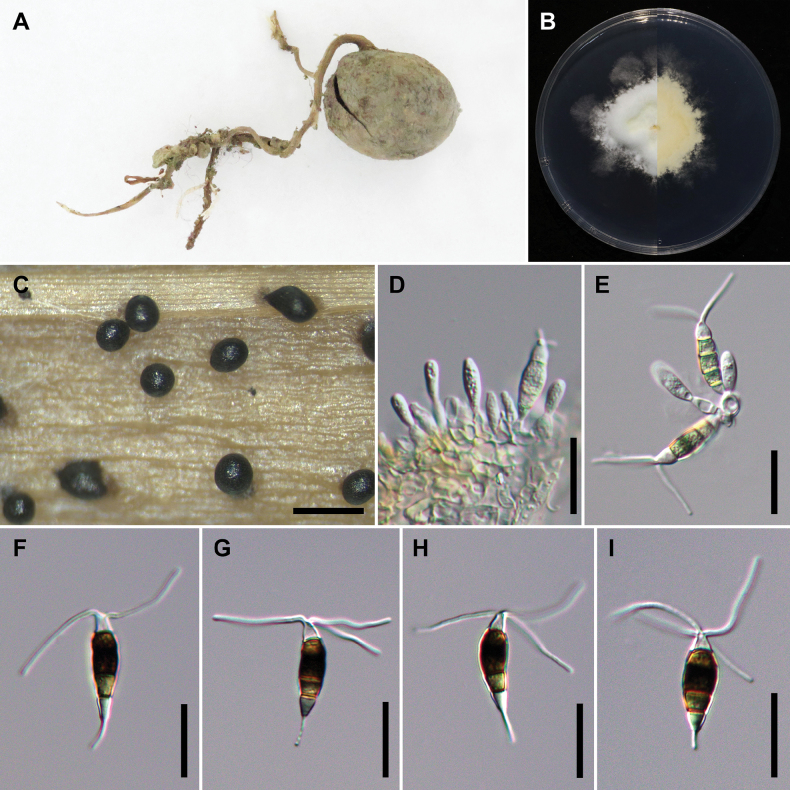
*Neopestalotiopsiscamelliae-oleiferae* (NTUPPMCC 18-166 = CD08) **A** the original habitat of *Neopestalotiopsiscamelliae-oleiferae*; the stroma of *Tolypocladium* sp. hyperparasitic on an ascocarps of *Elaphomyces* sp. (Ascomycota) **B** top view (left) and bottom view (right) of the colony on potato dextrose agar (PDA) after incubation for seven days **C** conidiomata on carnation leaf **D, E** conidiogenous cells and immature conidia **F–I** conidia. Scale bars: 250 μm (**C**); 20 μm (**D–I**).

##### Culture characteristics.

Colonies on PDA reaching 46.25 mm diam. on average after culturing at 25 °C in the dark for seven days, filamentous to circular, with slightly undulate edge, aerial mycelium dense, white to yellowish; reverse yellowish.

##### Materials examined.

Taiwan, New Taipei City, Sanxia District, Manyueyuan National Forest Recreation Area, on stroma of *Tolypocladium* sp. hyperparasitic on an ascocarps of *Elaphomyces* sp. (Ascomycota), 25 May 2018, Wei-Yu Chuang, living culture NTUPPMCC 18-166 (= CD08).

##### Notes.

*Neopestalotiopsiscamelliae-oleiferae* was originally documented by Li et al. (2021) and the isolate NTUPPMCC 18-166, used in the present study, share comparable morphological features with the illustration of holotype material (CSUFT 081). As a result, the present study recognised NTUPPMCC 18-166 as *N.camelliae-oleiferae*. Additionally, this marks the first report of *N.camelliae-oleiferae* in Taiwan.

#### ﻿ Neopestalotiopsis haikouensis

Taxon classificationFungiAmphisphaerialesSporocadaceae

Z.X. Zhang, J.W. Xia & X.G. Zhang, 2022

11A719A6-104F-5B53-B7E9-FCE0F3ABAC60

Fig. 9

##### Description.

On carnation leaves (*Dianthuscaryophyllus*) supplanted on WA (NTUPPMCC 18-163). Sexual morph was not observed in culture. Asexual morph: ***Conidiomata*** acervular, globose, semi-immersed, solitary or gregarious, 50–250 μm diam.; oozing globose, dark brown to black conidial masses. ***Conidiophores*** obclavate to subcylindrical, hyaline, smooth, annelidic, indistinct and frequently merged to conidiogenous cells. ***Conidiogenous cells*** ampulliform to subcylindrical, hyaline to pale brown, smooth, (2–)2.3–3.6(–5.2) × (5.4–)6.4–9(–10.1) μm, x– ± SD = 2.9 ± 0.7 × 7.7 ± 1.3 μm. ***Conidia*** fusoid to oval, straight or slightly curved, 4-septate, smooth, (4.7–)4.8–5.6(–6.4) × (22.6–)23.7–26.4(–28.5) μm, x– ± SD = 5.2 ± 0.4 × 25 ± 1.3 μm, bearing appendages; basal cell obconic with a truncate base, hyaline, thin-walled, (2.9–)4.3–5.5(–5.9) μm long, x– ± SD = 4.9 ± 0.6 μm; three median cells doliiform to subcylindrical, versicoloured, septa darker than the rest of the cell, thick-walled, the first median cell from base pale brown, (4.4–)4.6–5.4(–6.3) μm long (x– ± SD = 5 ± 0.4), the second median cell honey-brown to brown, (4.1–)4.4–5.2(–5.6) μm long (x– ± SD = 4.8 ± 0.4 μm), the third median cell brown, (4.6–)4.8–5.5(–5.9) μm long (x– ± SD = 5.2 ± 0.4 μm), together (13.4–)14.1–15.9(–17.5) μm long (x– ± SD = 15 ± 0.9 μm); apical cell conical to subcylindrical with a truncate or acute apex, hyaline, thick-walled, (3.5–)4.6–5.6(–6) μm long (x– ± SD = 5.1 ± 0.5 μm). ***Appendages*** tubular, hyaline, straight or slightly bent, apical appendage 2–3 (mostly 3), unbranched, (15.5–)17.7–25.5(–33.4) μm long (x– ± SD = 21.6 ± 3.9 μm), basal appendage single, centric, unbranched, (3.2–)4–6.3(–8.2) μm long (x– ± SD = 5.1 ± 1.2 μm).

**Figure 9.**
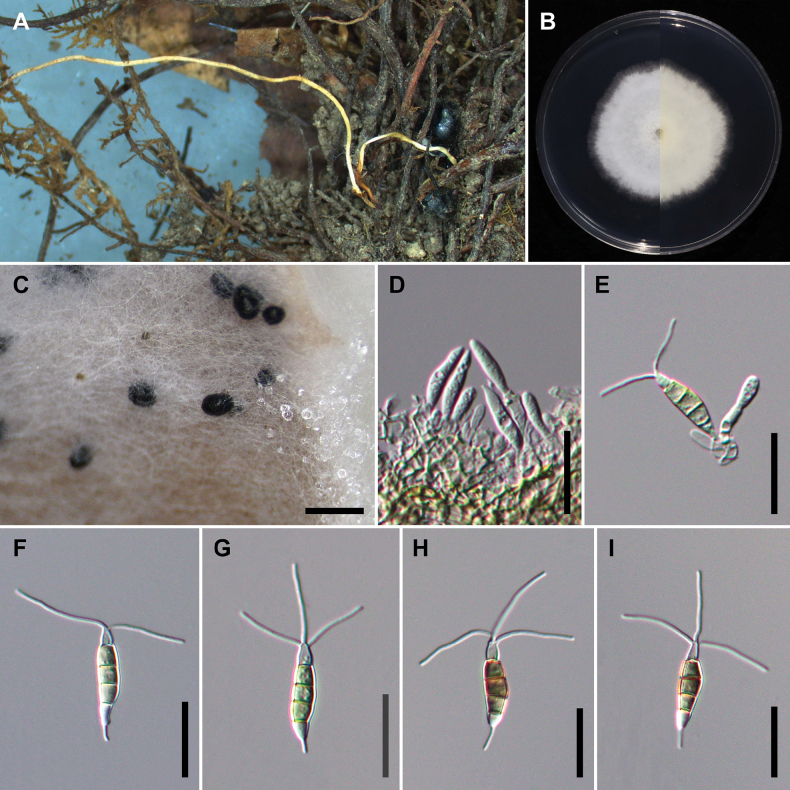
*Neopestalotiopsishaikouensis* (NTUPPMCC 18-163 = CD04) **A** the original habitat of *Neopestalotiopsishaikouensis*; the stroma of *Ophiocordyceps* sp. **B** top view (left) and bottom view (right) of the colony on potato dextrose agar (PDA) after incubation for seven days **C** conidiomata on carnation leaf **D, E** conidiogenous cells and immature conidia **F–I** conidia. Scale bars: 250 μm (**C**); 20 μm (**D–I**).

##### Culture characteristics.

Colonies on PDA reaching 45.65 mm diam. on average after culturing at 25 °C in the dark for seven days, filamentous to circular, flat with undulate edge, aerial mycelium moderate dense, white to grey white; reverse similar in colour.

##### Materials examined.

Taiwan, New Taipei City, Sanxia District, Manyueyuan National Forest Recreation Area, on stroma of *Ophiocordyceps* sp. parasitic on an insect (Hymenoptera), 1 August 2018, Wei-Yu Chuang, living culture NTUPPMCC 18-163 (= CD04). Taiwan, Yilan County, Fushan Botanical Garden, on stroma of *Ophiocordyceps* sp. parasitic on an insect (Hymenoptera), 19 July 2018, Wei-Yu Chuang, living culture NTUPPMCC 18-164 (= CD05). Taiwan, New Taipei City, Sanxia District, Manyueyuan National Forest Recreation Area, on stroma of *Tolypocladium* sp. hyperparasitic on an ascocarps of *Elaphomyces* sp. (Ascomycota), 15 July 2021, Yu-Chen Lin, living culture NTUPPMCC 21-057 (= CD12).

##### Notes.

Multi-locus phylogenetic analysis revealed that three newly-identified strains (NTUPPMCC 18-163, NTUPPMCC 18-164 and NTUPPMCC 21-057) form a clade closely associated with the ex-type strain of *N.haikouensis* SAUCC 212271, with robust statistical support (MLB = 100%, MPB = 96%, PP = 1.00). Additionally, the DNA sequences of ITS (100%), *tub2* (99.73%) and *tef1-α* (99.77%) genes of NTUPPMCC 18-163 closely resemble those of the ex-type strain of *N.haikouensis* (SAUCC 212271) and the morphological features of NTUPPMCC 18-163 align with the original taxonomic description in Zhang et al. (2022). Hence, based on both the phylogenetic analysis utilising DNA sequence data and morphological characteristics, strains NTUPPMCC 18-163, NTUPPMCC 18-164 and NTUPPMCC 21-057 were identified as *Neopestalotiopsishaikouensis*. To the best of our knowledge, this study marks the first report of *N.haikouensis* in Taiwan.

#### ﻿ Neopestalotiopsis

Taxon classificationFungiAmphisphaerialesSporocadaceae

sp.

4238C770-061F-5278-B3D9-926BE4CD5B80

Fig. 10

##### Description.

See Suppl. material 1: table S1.

##### Materials examined.

Taiwan, Pingtung County, Chunri Township, Tahan Forest Road, on stroma of *Beauveria* sp. parasitic on an insect (Lepidoptera), 7 October 2018, Wei-Yu Chuang, living culture NTUPPMCC 18-161 (= CD02).

**Figure 10.**
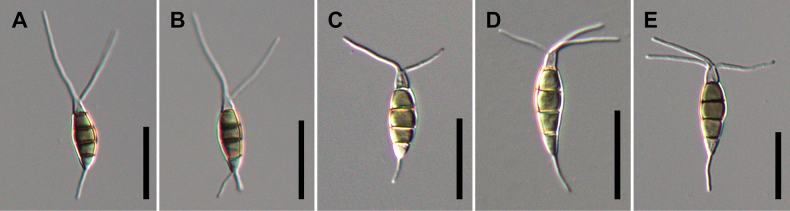
Conidial morphology of *Pestalotiopsisformosana* (**A, B** NTUPPMCC 21-056), *Pestalotiopsistrachycarpicola* (**C** NTUPPMCC 18-160 **D** NTUPPMCC 21-055) and *Neopestalotiopsis* sp. (**E** NTUPPMCC 18-161), isolated from entomopathogenic fungi in this study. Scale bars: 20 μm (**A–E**).

##### Notes.

As mentioned earlier in this manuscript, even though the new strain NTUPPMCC 18-161 formed a distinct clade basal to *N.asiatica*, *N.chrysea*, *N.macadamiae* and *N.umbrinospora* in all ML, MP and BI phylogenetic trees, based on the concatenated DNA sequence data matrix, it did not consistently form clades in most of single-locus trees. For instance, in the ITS phylogeny (Suppl. material 2: fig. S8), NTUPPMCC 18-161 clustered with *N.acrostichi* (MFLUCC 17-1755) and the clade containing the ex-type strain (MFLUCC 15-0776) of *N.musae*, along with two representative strains (MM3-2z9A and MM3-2z9C). Conversely, in the phylogenetic tree, based on *tub2* (Suppl. material 2: fig. S9), NTUPPMCC 18-161 formed a well-supported clade with the clade containing the ex-type strain of *N.asiatica* (MFLUCC 12-0286), *N.chrysea* (MFLUCC 12-0261), *N.coffeae-arabicae* (HGUP 4019), *N.macadamiae* (BRIP 63737c), *N.sonneratae* (MFLUCC 17-1744), *N.thailandica* (MFLUCC 17-1730) and *N.umbrinospora* (MFLUCC 12-0285). Meanwhile, it formed a separate sister clade to *N.guajavae*, *N.pandanicola* and *N.psidii* in the *tef1-α* phylogeny (Suppl. material 2: fig. S10) with poor branch and statistical support. When comparing the morphological features of strain NTUPPMCC 18-161 with its phylogenetically closelyrelated species, it becomes evident that our strain exhibits overlapping morphological features, particularly in the number of appendages and sizes of the conidial features (Suppl. material 1: table S10). Therefore, owing to the uncertainty in both phylogenetic placement and morphological data, we tentatively identify NTUPPMCC 18-161 as an unclassified *Neopestalotiopsis* strain. However, additional strains and information are required to clarify the correct placement of this strain.

### ﻿Growth rate

**Figure 11.**
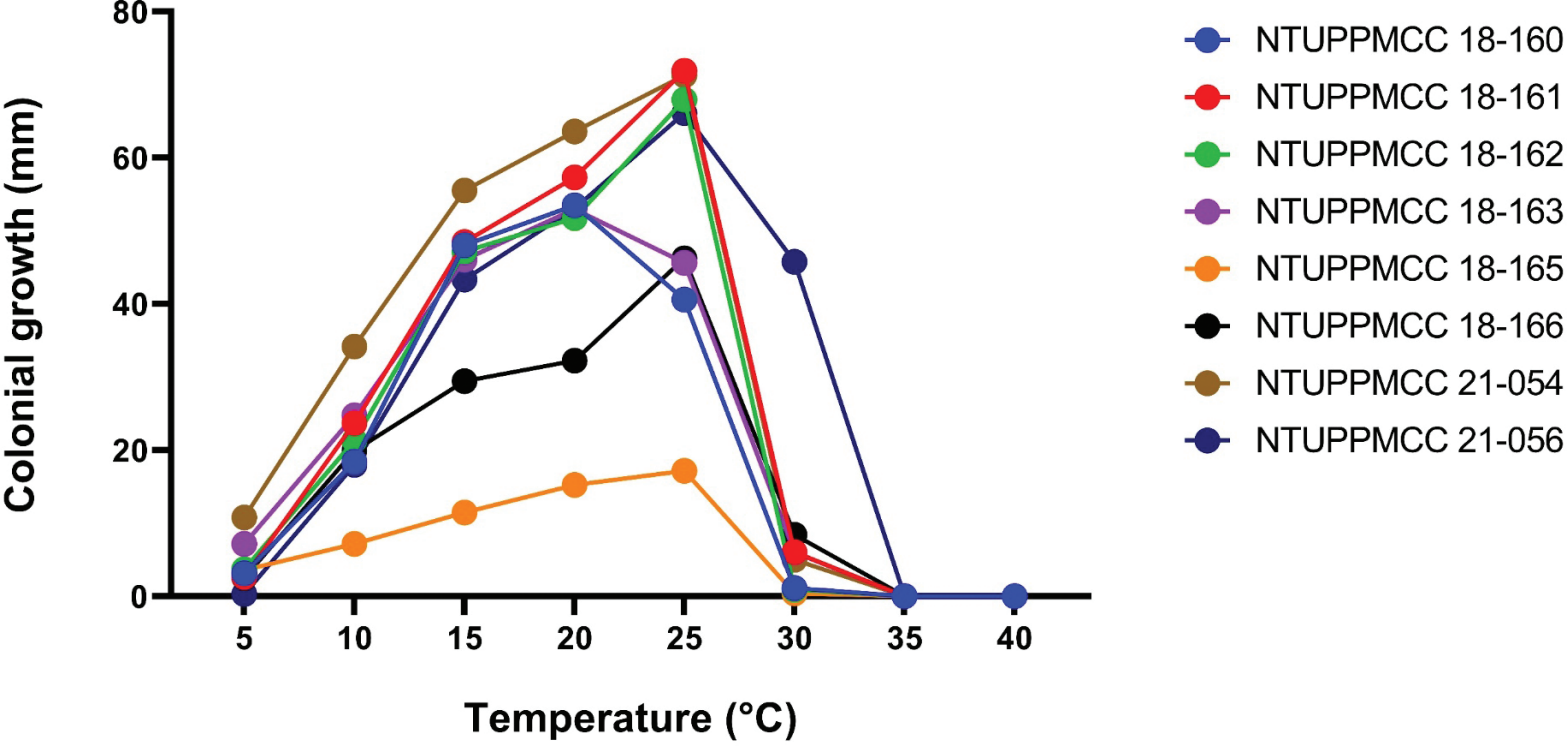
Temperature effect on mycelial growth according to the comparison of colonial growth (mm) of different species at each temperature, based on the mean values presented in Table S11. Colours represent different taxa: NTUPPMCC 18-160, *Pestalotiopsistrachycarpicola*; NTUPPMCC 18-161, *Neopestalotiopsis* sp.; NTUPPMCC 18-162, *Pestalotiopsishispanica*; NTUPPMCC 18-163, *Neopestalotiopsishaikouensis*; NTUPPMCC 18-165, *Pestalotiopsismanyueyuanani*.; NTUPPMCC 18-166, *Neopestalotiopsiscamelliae-oleiferae*; NTUPPMCC 21-054, *Pestalotiopsischamaeropis*; NTUPPMCC 21-056, *Pestalotiopsisformosana*.

Based on the results of the phylogenetic analysis, single strains representing each species were selected to test the growth rate. In total, eight strains were selected and grown on PDA media at 25 °C in the dark for seven days. The diameters of colonies were measured (mm) and the means were calculated and are shown in Fig. 11. Isolate NTUPPMCC 18-161 (*Neopestalotiopsis* sp.) and isolate NTUPPMCC 21-054 (*P.chamaeropis*) showed the widest diameter colonies (71.9 and 71.3 mm on average, respectively) and displayed significantly faster growth after seven days of incubation. In contrast, strain NTUPPMCC 18-165 (*P.manyueyuanani*) had the lowest colony diameter (17.15 mm on average) exhibiting significantly low growth compared to all the other isolates.

### ﻿Temperature effects

**Figure 12.**
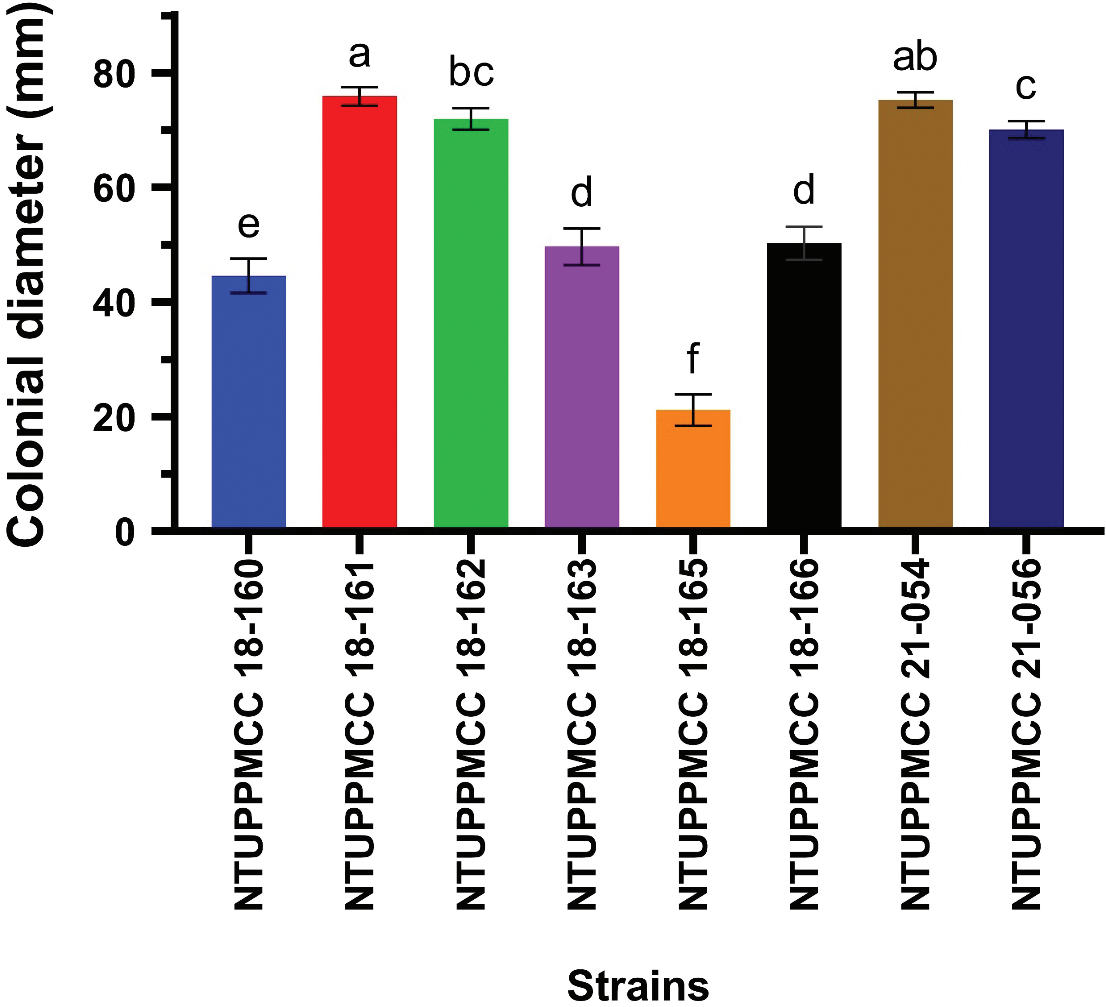
Comparison of the mycelium growth rates of eight pestalotiopsis-like fungal strains at 25 °C. According to Tukey’s range test, data (mean ± standard deviation) with the same letters are not significantly different. Colours represent different taxa: NTUPPMCC 18-160, *Pestalotiopsistrachycarpicola*; NTUPPMCC 18-161, *Neopestalotiopsis* sp.; NTUPPMCC 18-162, *Pestalotiopsishispanica*; NTUPPMCC 18-163, *Neopestalotiopsishaikouensis*; NTUPPMCC 18-165, *Pestalotiopsismanyueyuanani*.; NTUPPMCC 18-166, *Neopestalotiopsiscamelliae-oleiferae*; NTUPPMCC 21-054, *Pestalotiopsischamaeropis*; NTUPPMCC 21-056, *Pestalotiopsisformosana*.

Fungal mycelial growth was detected for all the tested isolates between 5 to 40 °C and measured as colony diameter. The results of the effect of temperatures on the mycelium growth of tested strains are presented in Fig. 11 and Suppl. material 1: table S11. The results showed that the temperature regimes intensely mediate the growth of tested fungal strains (*p* ≤ 0.05); the maximum growth was determined at 25 °C for most of the strains, except for NTUPPMCC 18-160 (*P.trachycarpicola*) and NTUPPMCC 18-163 (*N.haikouensis*) where it showed the highest growth at 20 °C (mean 53.5 mm and 52.95 mm). The results of comparison of the mycelium growth rates for all the representative strains at 25 °C were shown in Fig. 12. In addition, strain NTUPPMCC 19-165 (*P.manyueyuanani*) had no significant difference in mycelium growth between 20 °C and 25 °C, but both were significantly higher than other tested temperatures. However, for all the isolates (Fig. 11 and Suppl. material 1: table S11), the minimum or a lack of growth was observed at 35–40 °C.

### ﻿Optimal pH

**Figure 13.**
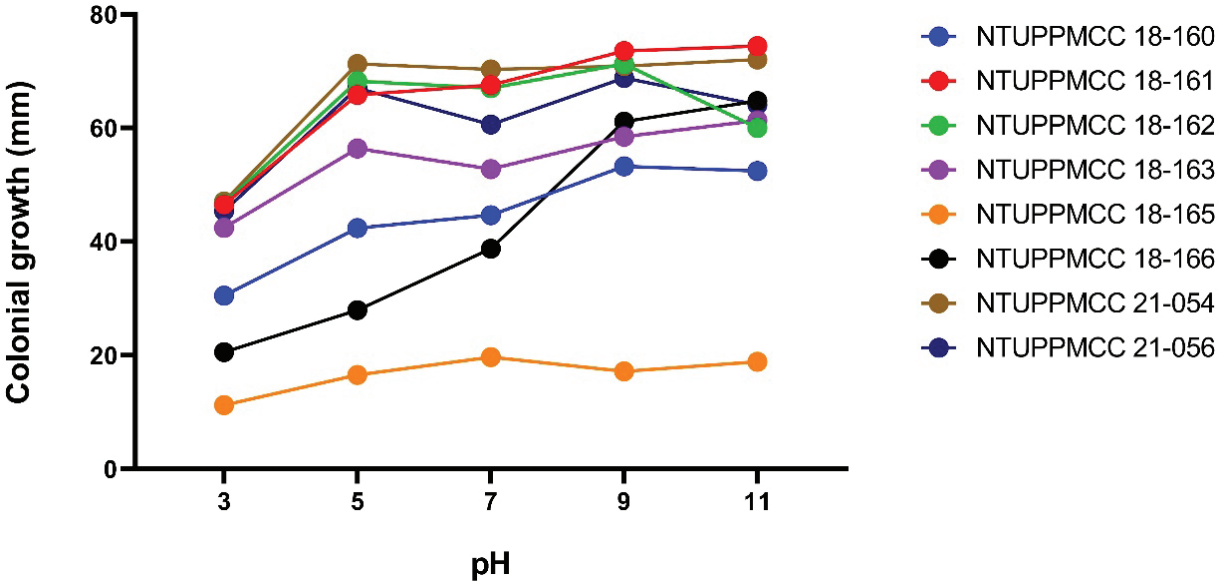
Optimal pH for mycelial growth of each species according to the comparison of colonial growth (mm) of different species at each pH, based on the mean values presented in Table S12. Colours represent different taxa: NTUPPMCC 18-160, *Pestalotiopsistrachycarpicola*; NTUPPMCC 18-161, *Neopestalotiopsis* sp.; NTUPPMCC 18-162, *Pestalotiopsishispanica*; NTUPPMCC 18-163, *Neopestalotiopsishaikouensis*; NTUPPMCC 18-165, *Pestalotiopsismanyueyuanani*.; NTUPPMCC 18-166, *Neopestalotiopsiscamelliae-oleiferae*; NTUPPMCC 21-054, *Pestalotiopsischamaeropis*; NTUPPMCC 21-056, *Pestalotiopsisformosana*.

The effect of pH on the mycelium growth of tested strains is shown in Fig. 13 and Suppl. material 1: table S12. Our results indicated that, generally, most of the strains used in this study grow better in alkaline medium (pH 7–11) compared with slightly acidic to neutral medium (pH 3–7). Except for isolate NTUPPMCC 21-056 (*P.formosana*), the maximum growth rates of isolates were at pH 5 and pH 9. Isolate NTUPPMCC 18-165 (*P.manyueyuanani*) showed relatively slow growth under all the tested pH upon mycelial growth compared with the other strains.

## ﻿Discussion

Species of *Pestalotiopsissensu lato* comprise a ubiquitous group of fungi that have been reported from various ecological niches. They have been identified as plant pathogens (Tsai et al. 2018, 2021; Fiorenza et al. 2022; Xiong et al. 2022; Zhang et al. 2022; Sun et al. 2023), human pathogens (Monden et al. 2013), saprobes (Ariyawansa and Hyde 2018; Sun et al. 2023) and endophytes (Maharachchikumbura et al. 2011; Sun et al. 2023). *Neopestalotiopsis* species have recently been identified as a group of emerging plant pathogens, causing severe diseases on economically important crops and fruits, such as strawberry (Baggio et al. 2021), guava (Solarte et al. 2018; Bhogal et al. 2022), grape (Huanaluek et al. 2021), mangosteen (Huanaluek et al. 2021), avocado (Fiorenza et al. 2022), blueberry (Santos et al. 2022), jabuticaba (Lin et al. 2022) and persimmon (Qin et al. 2023). Apart from that, many pestalotiopsis-like fungal species have been identified as promising in terms of producing novel biologically active compounds (Xie et al. 2014; Deshmukh et al. 2017). The highest diversity of pestalotiopsis-like fungi is recorded in tropical and subtropical countries (Jiang et al. 2022a; Peng et al. 2022; Xiong et al. 2022; Seifollahi et al. 2023; Sun et al. 2023). While most pestalotiopsis-like taxa are associated with plants (Maharachchikumbura et al. 2011), investigations regarding their biodiversity and occurrence in unusual habitats are rare (Liu et al. 2021; Rajulu et al. 2022).

In the present study, we identified several strains of *Pestalotiopsissensu lato*. The species grouped in *Pestalotiopsis* and *Neopestalotiopsis* were associated with the stromata of entomopathogenic fungal species, based on morphology coupled with evolutionary relationships obtained from multi-locus phylogeny. Classification of pestalotiopsis-like fungal species mainly relies on morphological features together with evolutionary relationships, based on the multi-locus phylogeny of ITS, *tub2* and *tef1-α* genes. Even though many species have been introduced in recent years using this criterion, we observed several inconsistencies in the species’ evolutionary relationships based on phylogeny. For example, the two strains. identified as *P.trachycarpicola* (NTUPPMCC 18-160 and NTUPPMCC 21-055) in the present study and the ex-type strains of *P.kenyana*, *P.oryzae* and *P.rhodomyrtus*, were clustered within the same clade of *P.trachycarpicola* containing the ex-type strain plus several representative strains (MFLU 18-2524 and NTUCC 18-004) included in ITS, *tub2* and multi-locus phylogenies (Fig. 2 and Suppl. material 2: figs S5–S7). A similar scenario was observed in the clade where one of the strains identified as *P.chamaeropis* (NTUPPMCC 21-056) in this study and the type strains *P.chamaeropis* (CBS 186.71) and *P.daliensis* (CGMCC 3.23548) clustered within the same clade in single gene and multi-locus phylogenies. In both scenarios, the trees have significantly short branch lengths and poor statistical support in BI, ML and MP analysis (Fig. 2, Suppl. material 2: figs S1–S2). Similar circumstances were detected in the topology of the *Neopestalotiopsis* phylogenetic tree. In the present study, both concatenated gene and single gene trees of *Neopestalotiopsis* resolved unstable topologies with poor branch lengths and low bootstrap support (Fig. 3, Suppl. material 2: figs S3, S4, S8–S10), consistent with previous studies (Maharachchikumbura et al. 2014; Liu et al. 2017; Liu et al. 2019; Tsai et al. 2021). Hence, it is essential to conduct further investigations to ascertain whether the limited informative loci result in an unambiguously resolved phylogram or if the poorly-resolved branches signify populations rather than distinct species.

We also implemented Genealogical Concordance Phylogenetic Species Recognition (GCPSR) to understand the species limits of *Pestalotiopsis* and *Neopestalotiopsis* taxa. This approach has been applied to delineate species in several fungal groups (Dettman et al. 2003; Tsai et al. 2018, 2021; Hilário et al. 2021a, 2021b). However, lacking DNA sequence data of some gene regions of ex-type strains, such as *P.brassicae*, *P.chinensis*, *P.dianellae*, *P.digitalis*, *P.dracontomelonis*, *P.eleutherococci*, *P.endophytica*, *P.hainanensis*, *P.kunmingensis*, *P.sequoiae*, *P.unicolor*, *P.verruculosa* and *P.yunnanensis*, plus the absence of type strains for some important species, such as *P.disseminata* and *P.longiseta* (Maharachchikumbura et al. 2012), meant that this approach was not useful to delimit species. Thus, future studies are essential to provide the correct natural classification of these taxa by providing missing sequence data and applying recently proposed DNA-based delimitation techniques, such as Generalised Mixed Yule Coalescent (GBH) (Fujisawa and Barraclough 2013) or Poisson Tree Processes (PTP) (Zhang et al. 2013).

Environmental factors including temperature and pH are the most important components mediating fungal growth and helping researchers understand the biology of fungal taxa. In general, pestalotiopsis-like fungi show optimal growth at 20–30 °C (Liu 1995; Tsukamoto and Hino 1956; Hopkins and McQuilken 2000; Liu et al. 2021). Most of the known pestalotiopsis-like fungi typically have a wide pH optimum, regularly covering 5–9 pH units without substantial inhibition of their growth (Wheeler et al. 1991; Nevarez et al. 2009; Das et al. 2010). In the present study, all strains used in the *in-vitro* mycelial growth rate test under different temperatures demonstrated high radial mycelia growth at 25 °C, consistent with previous studies (Tsukamoto and Hino 1956; Liu 1995). Conversely, most of the strains used in the present study showed optimal mycelial growth under alkaline conditions (pH 7–11). These observations somewhat differ from previous findings (pH 5–7) regarding the species in *Pestalotiopsissensu lato* (Tsukamoto and Hino 1956; Hopkins and McQuilken 2000; Liu et al. 2021).

In recent years, various studies have illustrated novel and promising taxa associated with stromata of entomopathogenic fungi, such as *Cordyceps* and *Ophiocordyceps*. For instance, Ariyawansa et al. (2018a) introduced a new pleosporalean family Tzeananiaceae typified by the genus *Tzeanania* and the species *T.taiwanensis* to accommodate a strain isolated from mycelium growing on the stroma of an *Ophiocordyceps* species. In addition, Sun et al. (2016) illustrated a novel pathogenic taxon, which causes significant quality and yield losses, from the stromata of *Cordycepsmilitaris* and named *Calcarisporiumcordycipiticola*. Although in the present study a single conidia culture of pestalotiopsis-like fungi was obtained from the stromata of entomopathogenic fungal taxa and their morphological features were observed, based on the spore-bearing structures formed on CLA, single spore isolation of their hosts, entomopathogenic fungal strains, were not successful. Therefore, we could not elucidate the potential nutritional mode of these pestalotiopsis-like fungal isolates or interactions with their entomopathogenic fungal hosts. Hence, further investigations are vital to understanding the interaction between these unusual fungal strains and their hosts.

## ﻿Conclusions

The present study introduced a novel species, *Pestalotiopsismanyueyuanani* and reported four new records *N.camelliae-oleiferae*, *N.haikouensis*, *P.chamaeropis* and *P.hispanica* in Taiwan for the first time. The optimal temperature for *in-vitro* mycelial growth in selected strains from these taxa was found to be 25 °C. Growth was observed to cease at both 5 °C and 35 °C. Furthermore, all strains exhibited faster growth under alkaline conditions when compared to acidic or neutral pH environments. This study expands our knowledge of the diversity of pestalotiopsis-like fungi in Taiwan. Additionally, it represents the first assessment of pestalotiopsis-like fungi associated with the stromata of entomopathogenic fungal taxa. However, since none of the entomopathogenic fungi in this study was successfully isolated and cultured, interactions between these pestalotiopsis-like fungi and entomopathogenic fungi are still unknown.

## Supplementary Material

XML Treatment for Pestalotiopsis chamaeropis

XML Treatment for Pestalotiopsis formosana

XML Treatment for Pestalotiopsis hispanica

XML Treatment for Pestalotiopsis manyueyuanani

XML Treatment for Pestalotiopsis trachycarpicola

XML Treatment for Neopestalotiopsis camelliae-oleiferae

XML Treatment for Neopestalotiopsis haikouensis

XML Treatment for Neopestalotiopsis

## ﻿Additional information

### Author ORCIDs

Sheng-Yu Hsu https://orcid.org/0000-0001-6227-0936

Yuan-Cheng Xu https://orcid.org/0000-0002-3885-498X

Yu-Chen Lin https://orcid.org/0000-0002-2319-0869

Marc Stadler https://orcid.org/0000-0002-7284-8671

Narumon Tangthirasunun https://orcid.org/0000-0001-7619-9464

Ratchadawan Cheewangkoon https://orcid.org/0000-0001-8576-3696

Hind A. AL-Shwaiman https://orcid.org/0009-0002-5872-9083

Abdallah M. Elgorban https://orcid.org/0000-0003-3664-7853

Hiran A. Ariyawansa https://orcid.org/0000-0001-8526-7721

### Data availability

All of the data that support the findings of this study are available in the main text or Supplementary Information.

## Supplementary materials

Supplementary material 1 The morphological features of *Pestalotiopsis**sensu lato* fungi and GenBank accession numbers Data type xlsx Explanation note **table S1.** The morphological features of pestalotiopsis-like fungi isolated in this study; **table S2.** GenBank accession numbers of *Pestalotiopsis* isolates included in the phylogenetic analysis; **table S3.** GenBank accession numbers of *Neopestalotiopsis* isolates included in the phylogenetic analysis; **table S4.** Primers and PCR conditions used to amplify ITS, *tub2* and *tef1-α* gene regions; **table S5.** Nucleotide substitution models used in the phylogenetic analyses; **table S6.** Morphological comparison of *Pestalotiopsischamaeropis* and its closely-related taxa; **table S7.** Morphological comparison of *Pestalotiopsishispanica* and its closely-related taxa; **table S8.** Morphological comparison of *Pestalotiopsismanyueyuanani* and its closely-related taxa; **table S9.** Morphological comparison of *Pestalotiopsistrachycarpicola* and its closely-related taxa; **table S10.** The morphological comparison of *Neopestalotiopsis* strain NTUPPMCC 18-161 and its closely-related taxa; **table S11**. Mycelium growth of individual isolate of pestalotiopsis-like fungi at each temperature in this study; **table S12.** Mycelium growth of individual isolates of pestalotiopsis-like fungi at each pH in this study. https://binary.pensoft.net/file/976801This dataset is made available under the Open Database License (http://opendatacommons.org/licenses/odbl/1.0/). The Open Database License (ODbL) is a license agreement intended to allow users to freely share, modify, and use this Dataset while maintaining this same freedom for others, provided that the original source and author(s) are credited.Sheng-Yu Hsu, Yuan-Cheng Xu, Yu-Chen Lin, Wei-Yu Chuang, Shiou-Ruei Lin, Marc Stadler, Narumon Tangthirasunun, Ratchadawan Cheewangkoon, Hind A. AL-Shwaiman, Abdallah M. Elgorban, Hiran A. Ariyawansa

Supplementary material 2 Phylogenetic trees generated by maximum parsimony analysis of single and combined ITS, *tub2* and *tef1-α* sequence data Data type pdf Explanation note **fig. S1.** Phylogenetic tree generated by maximum parsimony analysis of combined ITS, *tub2* and *tef1-α* sequence data of *Pestalotiopsis*. MPB values ≥ 70% are given at the nodes. The scale-bar represents the number of nucleotide substitutions per site. *Neopestalotiopsisprotearum* CBS 114178 was used as an outgroup to rooting the tree. New isolates are in red and taxa representing ex-type cultures are in bold; **fig. S2.** Phylogenetic tree obtained through Bayesian Inference for the dataset of ITS, *tub2* and *tef1-α* loci of *Pestalotiopsis*. PP ≥ 0.95 are given at the nodes. The scale-bar represents the number of nucleotide substitutions per site. *Neopestalotiopsisprotearum* CBS 114178 was used as an outgroup for rooting the tree. New isolates are in red and taxa representing ex-type cultures are in bold; **fig. S3.** Phylogenetic tree generated by maximum parsimony analysis of combined ITS, *tub2* and *tef1-α* sequence data of *Neopestalotiopsis*. MPB values ≥ 70% are given at the nodes. The scale-bar represents the number of nucleotide substitutions per site. *Pseudopestalotiopsistheae* MFLUCC 12-0055 was used as an outgroup for rooting the tree. New isolates are in red and taxa representing ex-type cultures are in bold; **fig. S4.** Phylogenetic tree obtained through Bayesian inference for the dataset of ITS, *tub2* and *tef1-α* loci of *Neopestalotiopsis*. PP ≥ 0.95 are given at the nodes. The scale-bar represents the number of nucleotide substitutions per site. *Pseudopestalotiopsistheae* MFLUCC 12-0055 was used as an outgroup for rooting the tree. New isolates are in red and taxa representing ex-type cultures are in bold; **fig. S5.** Phylogenetic tree generated by Maximum Likelihood analysis of ITS sequence data of *Pestalotiopsis*. MLB values ≥ 70% are given at the nodes. The scale-bar shows the number of estimated substitutions per site. *Neopestalotiopsisprotearum* (CBS 114178) was used as an outgroup for rooting the tree. New isolates are in red and taxa representing ex-type cultures are in bold; **fig. S6.** Phylogenetic tree generated by Maximum Likelihood analysis of *tub2* sequence data of *Pestalotiopsis*. MLB values ≥ 70% are given at the nodes. The scale-bar shows the number of estimated substitutions per site. *Neopestalotiopsisprotearum* (CBS 114178) was used as an outgroup for rooting the tree. New isolates are in red, and taxa representing ex-type cultures are in bold; **fig. S7.** Phylogenetic tree generated by Maximum Likelihood analysis of *tef1-α* sequence data of *Pestalotiopsis*. MLB values ≥ 70% are given at the nodes. The scale-bar shows the number of estimated substitutions per site. *Neopestalotiopsisprotearum* (CBS 114178) was used as an outgroup for rooting the tree. New isolates are in red and taxa representing ex-type cultures are in bold; **fig. S8.** Phylogenetic tree generated by Maximum Likelihood analysis of ITS sequence data of *Neopestalotiopsis*. MLB values ≥ 70% are given at the nodes. The scale-bar shows the number of estimated substitutions per site. *Pseudopestalotiopsistheae* MFLUCC 12-0055 was used as an outgroup for rooting the tree. New isolates are in red and taxa representing ex-type cultures are in bold; **fig. S9.** Phylogenetic tree generated by Maximum Likelihood analysis of *tub2* sequence data of *Neopestalotiopsis*. MLB values ≥ 70% are given at the nodes. The scale-bar shows the number of estimated substitutions per site. *Pseudopestalotiopsistheae* MFLUCC 12-0055 was used as an outgroup for rooting the tree. New isolates are in red and taxa representing ex-type cultures are in bold; **fig. S10.** Phylogenetic tree generated by Maximum Likelihood analysis of *tef1-α* sequence data of *Neopestalotiopsis*. MLB values ≥ 70% are given at the nodes. The scale-bar shows the number of estimated substitutions per site. *Pseudopestalotiopsistheae* MFLUCC 12-0055 was used as an outgroup for rooting the tree. New isolates are in red and taxa representing ex-type cultures are in bold. https://binary.pensoft.net/file/976802This dataset is made available under the Open Database License (http://opendatacommons.org/licenses/odbl/1.0/). The Open Database License (ODbL) is a license agreement intended to allow users to freely share, modify, and use this Dataset while maintaining this same freedom for others, provided that the original source and author(s) are credited.Sheng-Yu Hsu, Yuan-Cheng Xu, Yu-Chen Lin, Wei-Yu Chuang, Shiou-Ruei Lin, Marc Stadler, Narumon Tangthirasunun, Ratchadawan Cheewangkoon, Hind A. AL-Shwaiman, Abdallah M. Elgorban, Hiran A. Ariyawansa
