# Supplementary material for: ﻿Hidden diversity of Pestalotiopsis and Neopestalotiopsis (Amphisphaeriales, Sporocadaceae) species allied with the stromata of entomopathogenic fungi in Taiwan
Source: MycoKeys. 2024 Jan 31;101:275–312. doi: 10.3897/mycokeys.101.113090 (PMC10851163; doi:10.3897/mycokeys.101.113090)
Supplement: Supplementary material 2 — Phylogenetic trees generated by maximum parsimony analysis of single and combined ITS, tub2 and tef1-α sequence data [file mycokeys-101-275-s002.pdf]

Figure S1. Phylogenetic tree generated by maximum parsimony analysis of combined ITS, *tub2*, and *tef1-α* sequence data of *Pestalotiopsis*. MPB values ≥ 70% are given at the nodes. The scale bar represents the number of nucleotide substitutions per site. *Neopestalotiopsis protearum* CBS 114178 was used as an outgroup to rooting the tree. New isolates are in red and taxa representing ex-type cultures are in bold.

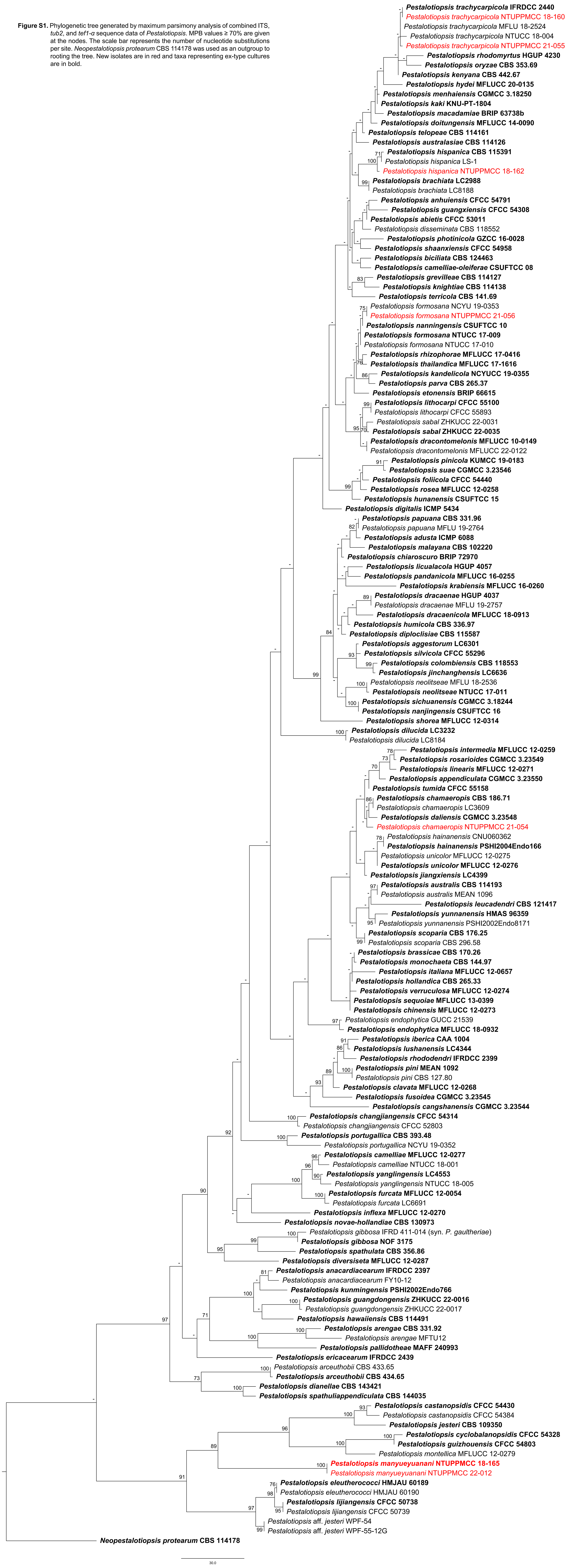

Figure S2. Phylogenetic tree obtained through Bayesian inference for the dataset of ITS, *tub2*, and *tef1-α* loci of *Pestalotiopsis*. PP ≥ 0.95 are given at the nodes. The scale bar represents the number of nucleotide substitutions per site. *Neopestalotiopsis protearum* CBS 114178 was used as an outgroup for rooting the tree. New isolates are in red, and taxa representing ex-type cultures are in bold.

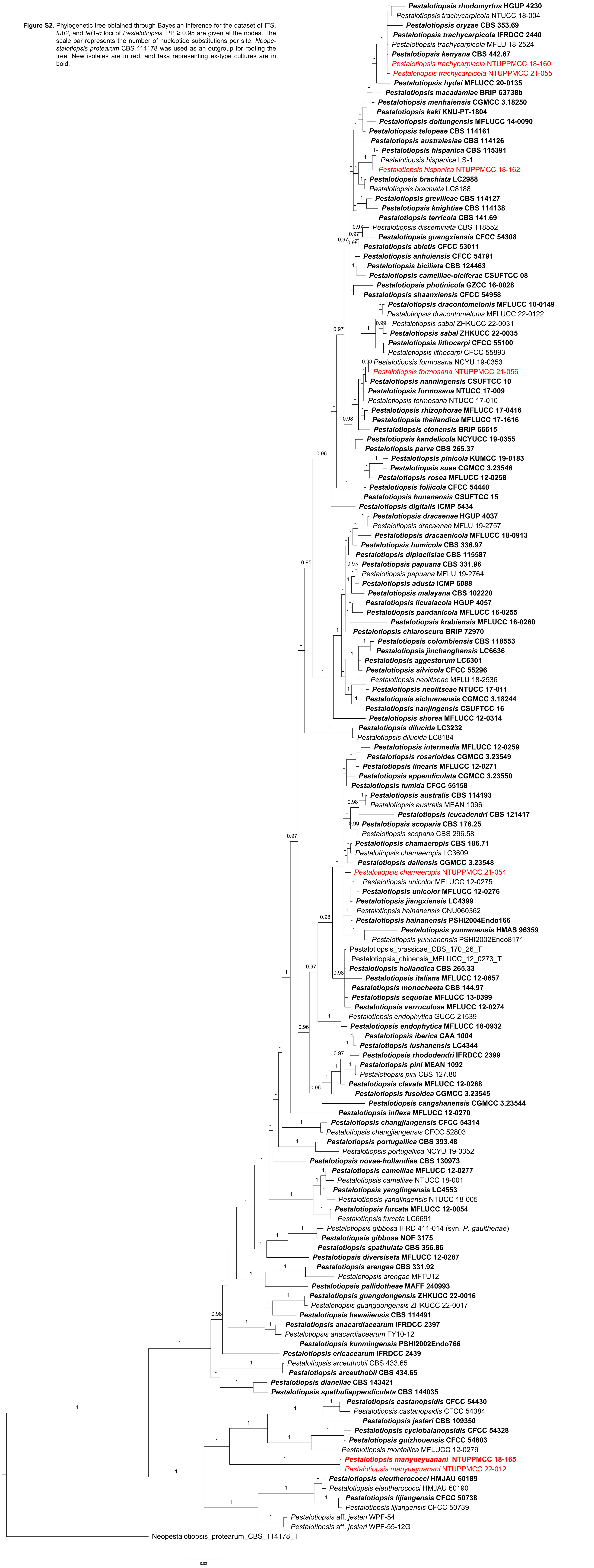

Figure S3. Phylogenetic tree generated by maximum parsimony analysis of combined ITS, *tub2*, and *tef1-α* sequence data of *Neopestalotiopsis*. MPB values ≥ 70% are given at the nodes. The scale bar represents the number of nucleotide substitutions per site. *Pseudopestalotiopsis theae* MFLUCC 12-0055 was used as an outgroup for rooting the tree. New isolates are in red, and taxa representing ex-type cultures are in bold.

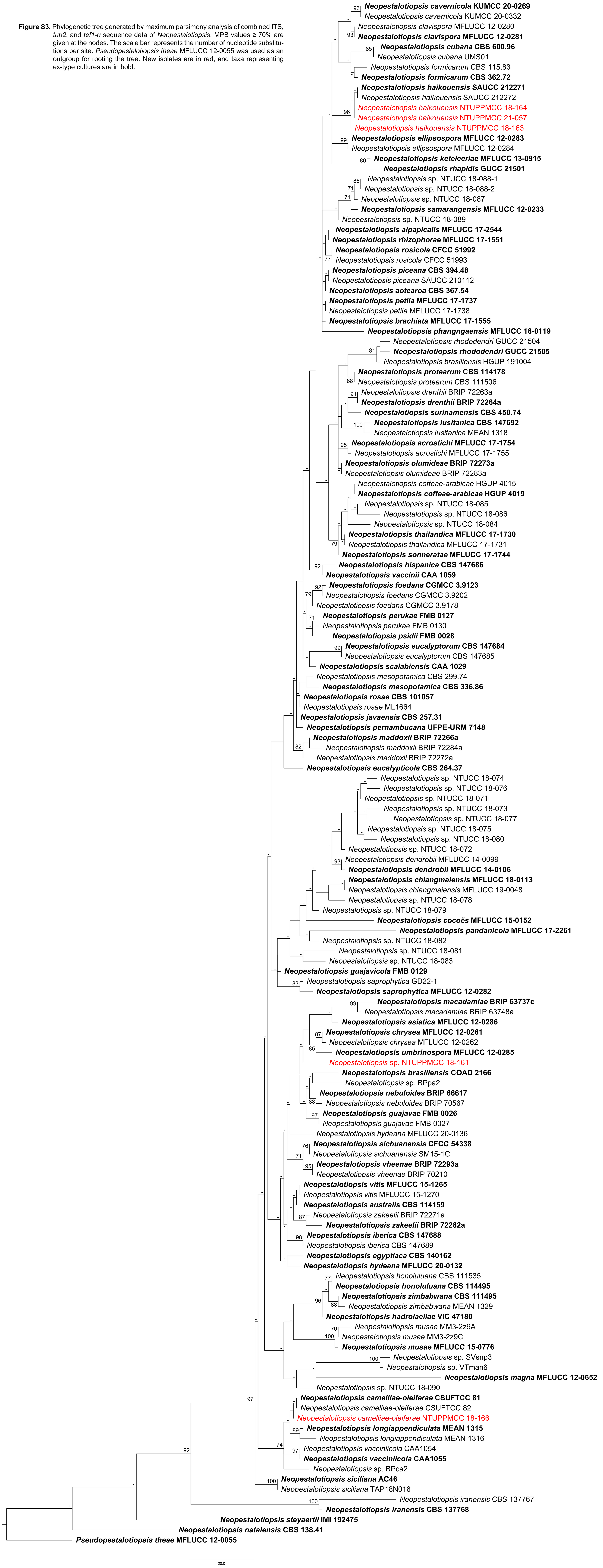

Figure S4. Phylogenetic tree obtained through Bayesian inference for the dataset of ITS, *tub2*, and *tef1-α* loci of *Neopestalotiopsis*. PP ≥ 0.95 are given at the nodes. The scale bar represents the number of nucleotide substitutions per site. *Pseudopestalotiopsis theae* MFLUCC 12-0055 was used as an outgroup for rooting the tree. New isolates are in red, and taxa representing ex-type cultures are in bold.

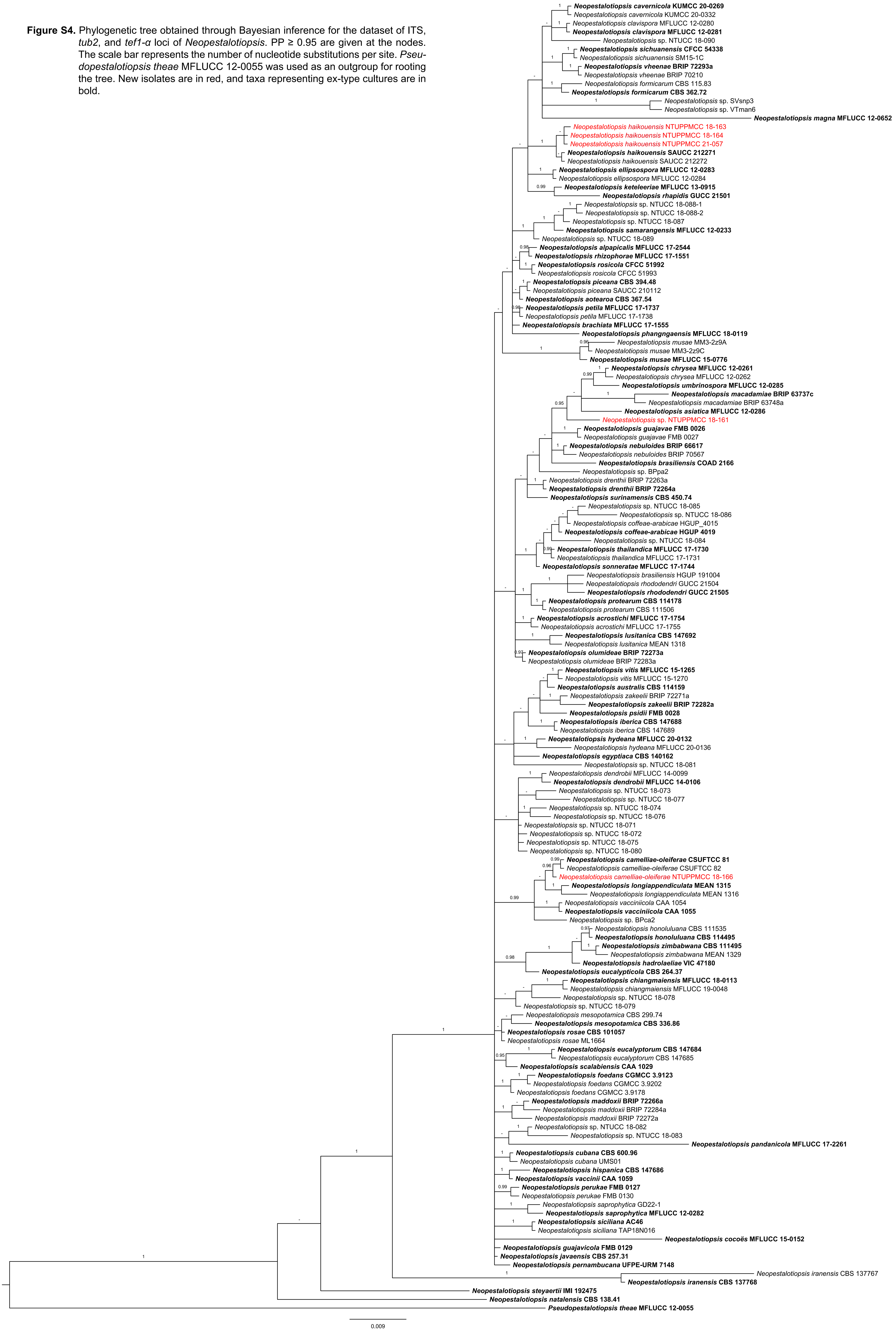

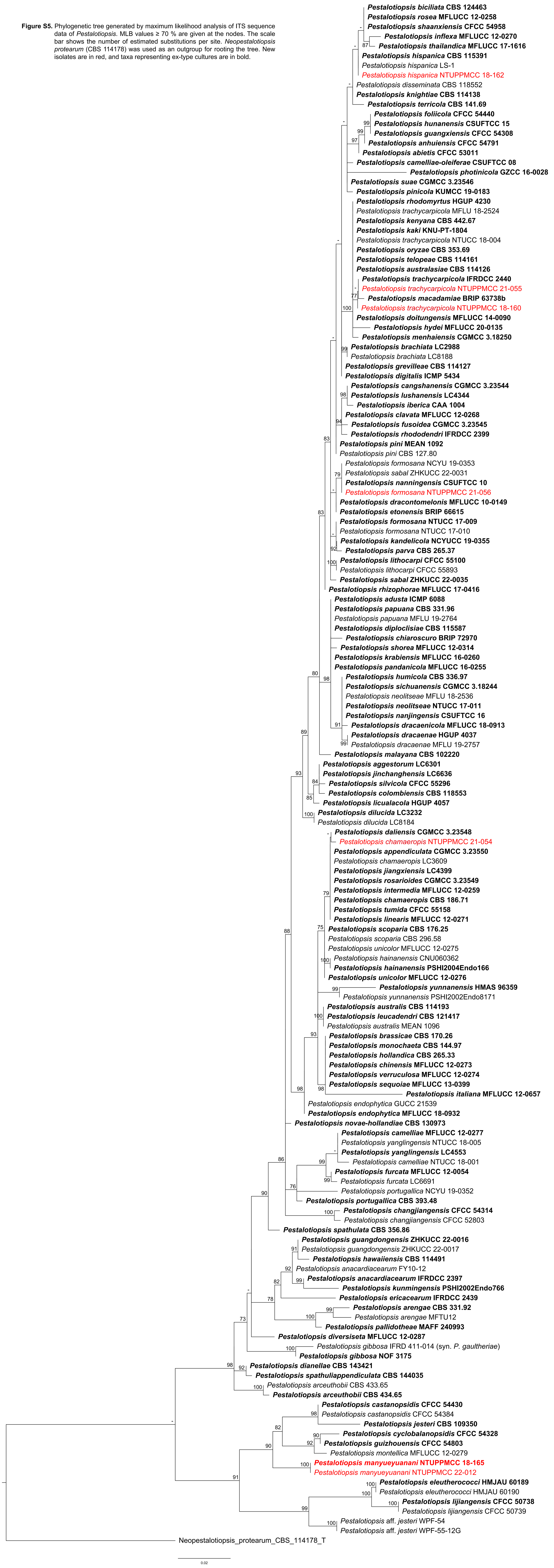

Figure S5. Phylogenetic tree generated by maximum likelihood analysis of ITS sequence data of *Pestalotiopsis*. MLB values ≥ 70 % are given at the nodes. The scale bar shows the number of estimated substitutions per site. *Neopestalotiopsis protearum* (CBS 114178) was used as an outgroup for rooting the tree. New isolates are in red, and taxa representing ex-type cultures are in bold.

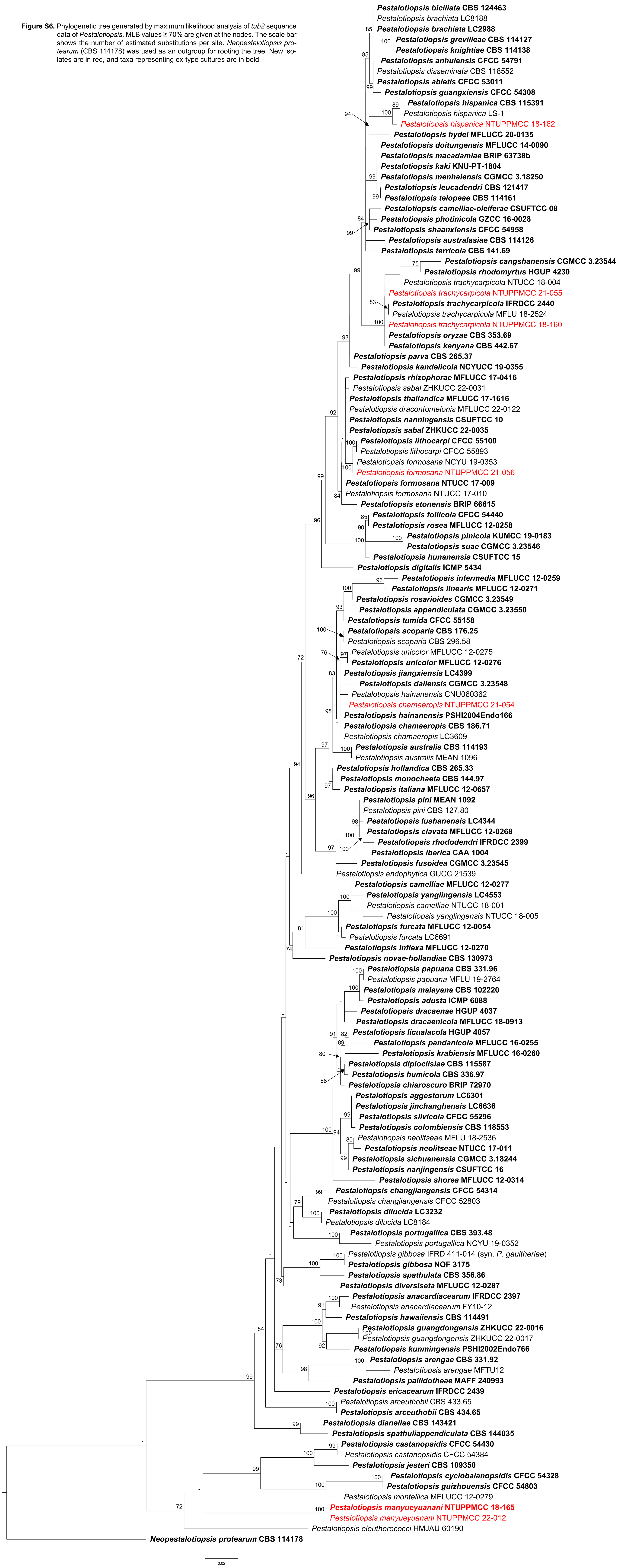

Figure S6. Phylogenetic tree generated by maximum likelihood analysis of *tub2* sequence data of *Pestalotiopsis*. MLB values ≥ 70% are given at the nodes. The scale bar shows the number of estimated substitutions per site. *Neopestalotiopsis protearum* (CBS 114178) was used as an outgroup for rooting the tree. New isolates are in red, and taxa representing ex-type cultures are in bold.

Figure S7. Phylogenetic tree generated by maximum likelihood analysis of *tef1-α* sequence data of *Pestalotiopsis*. MLB values ≥ 70% are given at the nodes. The scale bar shows the number of estimated substitutions per site. *Neopestalotiopsis protearum* (CBS 114178) was used as an outgroup for rooting the tree. New isolates are in red and taxa representing ex-type cultures are in bold.

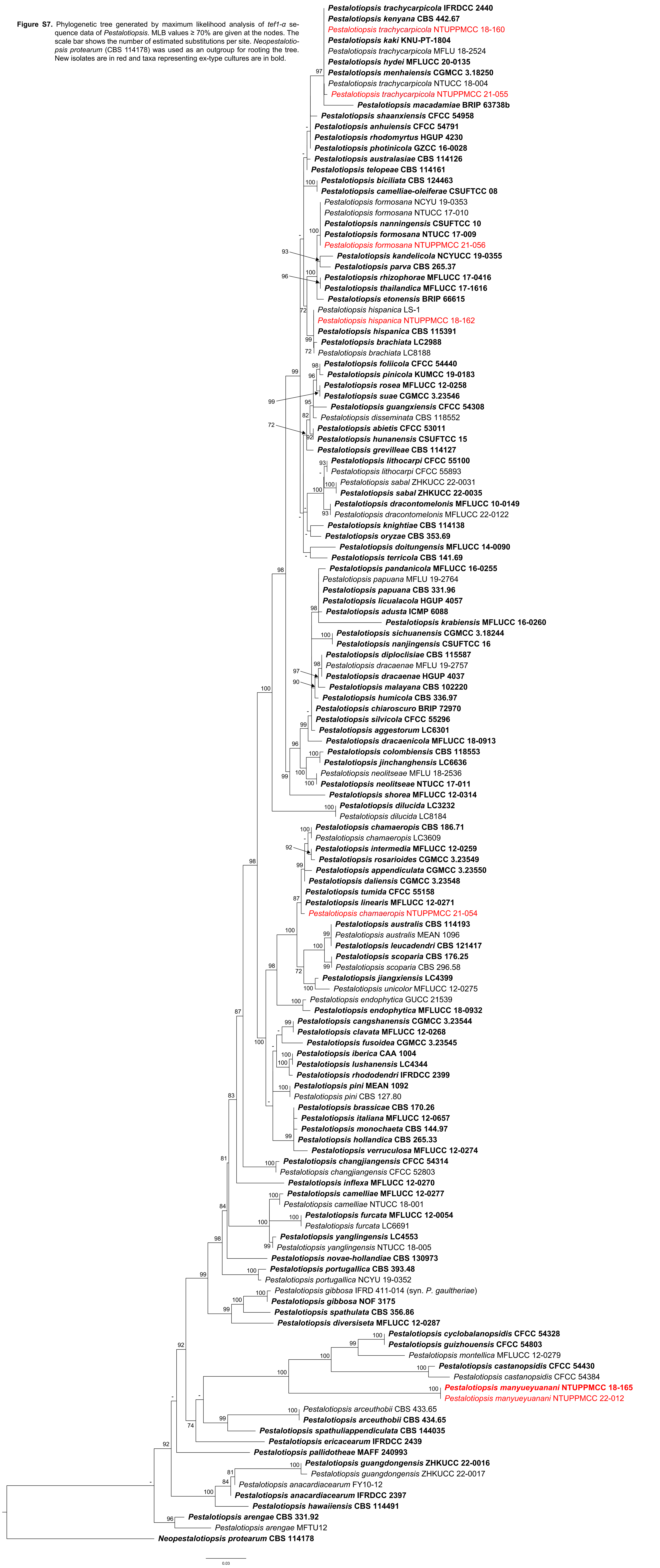

Figure S8. Phylogenetic tree generated by maximum likelihood analysis of ITS sequence data of *Neopestalotiopsis*. MLB values ≥ 70% are given at the nodes. The scale bar shows the number of estimated substitutions per site. *Pseudopestalotiopsis theae* MFLUCC 12-0055 was used as an outgroup for rooting the tree. New isolates are in red, and taxa representing ex-type cultures are in bold.

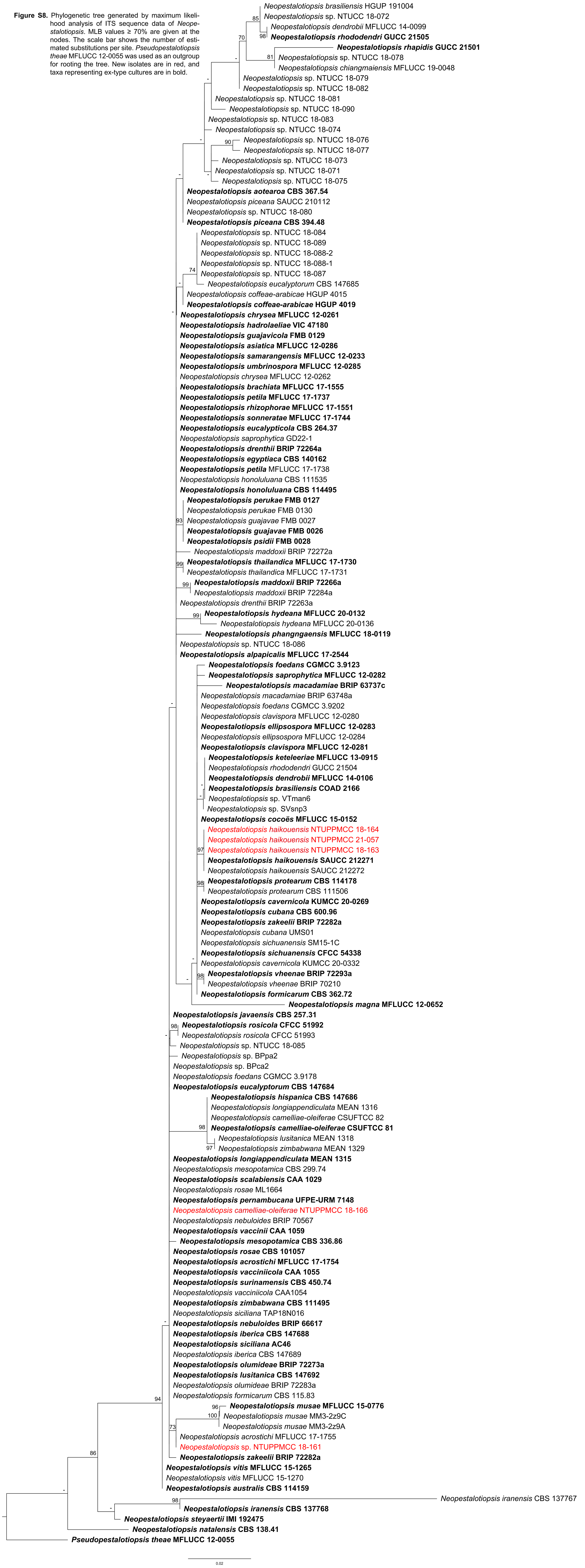

Figure S9. Phylogenetic tree generated by maximum likelihood analysis of *tub2* sequence data of *Neopestalotiopsis*. MLB values ≥ 70% are given at the nodes. The scale bar shows the number of estimated substitutions per site. *Pseudopestalotiopsis theae* MFLUCC 12-0055 was used as an outgroup for rooting the tree. New isolates are in red and taxa representing ex-type cultures are in bold.

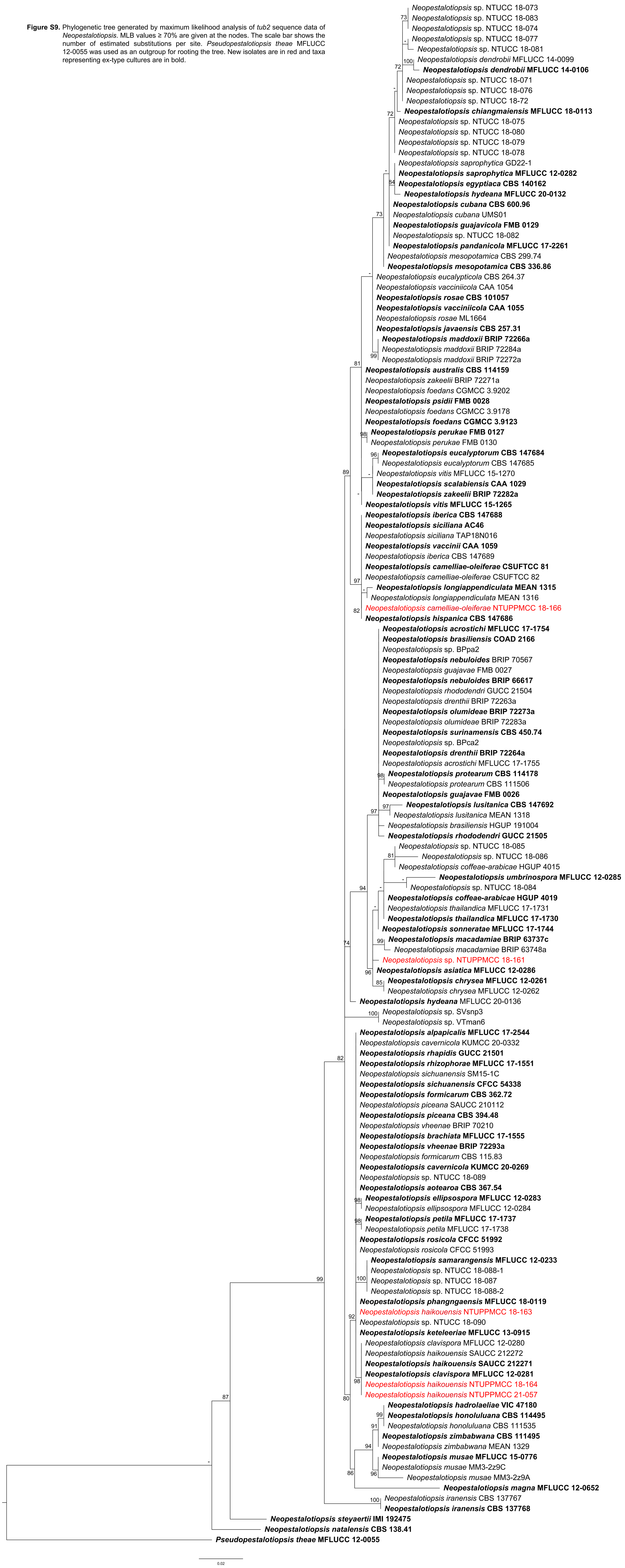

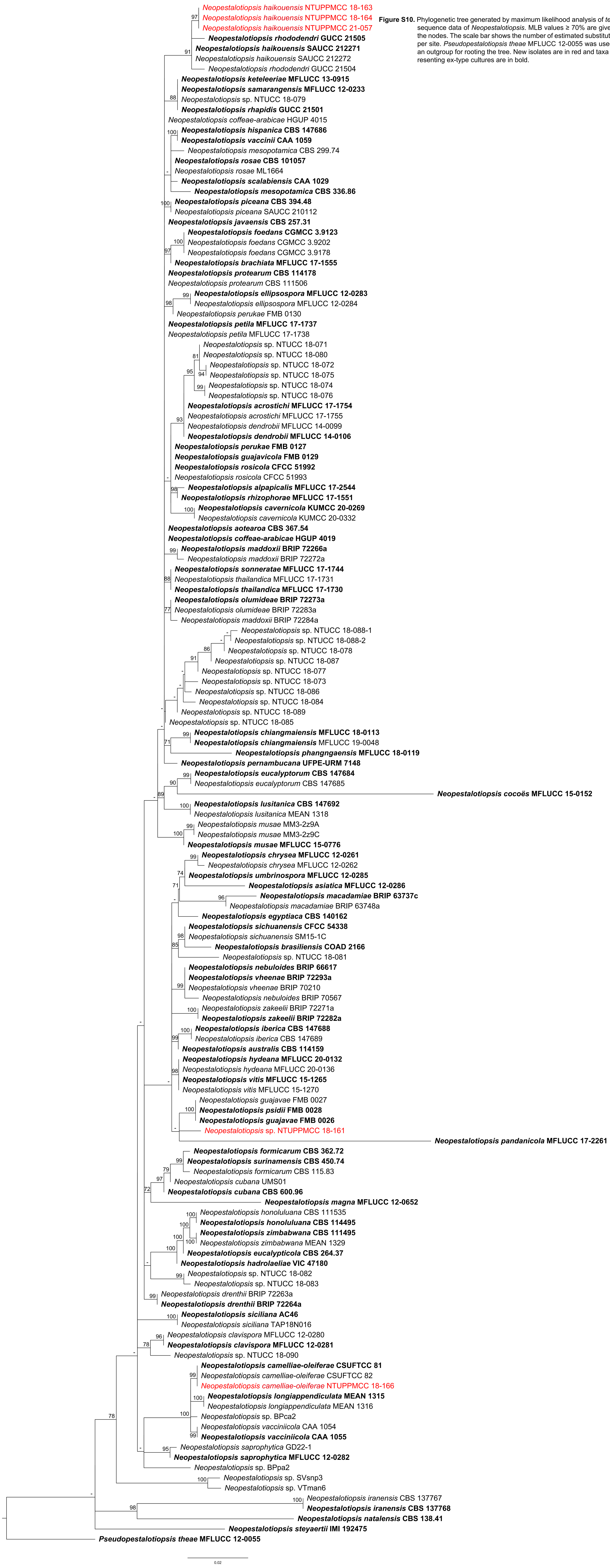

Figure S10. Phylogenetic tree generated by maximum likelihood analysis of *tef1-α* sequence data of *Neopestalotiopsis*. MLB values ≥ 70% are given at the nodes. The scale bar shows the number of estimated substitutions per site. *Pseudopestalotiopsis theae* MFLUCC 12-0055 was used as an outgroup for rooting the tree. New isolates are in red and taxa representing ex-type cultures are in bold.
